# V for vaccines and variants

**DOI:** 10.1007/s00191-023-00818-6

**Published:** 2023-06-01

**Authors:** Domenico Delli Gatti, Severin Reissl, Enrico Turco

**Affiliations:** 1 Department of Economics and Finance, Catholic University, Milan, Italy; 2 Complexity Lab in Economics, Catholic University, Milan, Italy; 3 CESifo, Munich, Germany; 4 RFF-CMCC European Institute on Economics and the Environment, Milan, Italy; 5 Fondazione Eni Enrico Mattei, Milan, Italy

**Keywords:** Agent-based models, Epidemic, Covid, Vaccination, Variant, E21, E22, E24, E27, I12, I15, I18

## Abstract

In the context of the Covid-19 pandemic, we evaluate the effects of vaccines and virus variants on epidemiological and macroeconomic outcomes by means of Monte Carlo simulations of a macroeconomic-epidemiological agent-based model calibrated using data from the Lombardy region of Italy. From simulations we infer that vaccination plays the role of a *mitigating factor*, reducing the frequency and the amplitude of contagion waves and significantly improving macroeconomic performance with respect to a scenario without vaccination. The emergence of a variant, on the other hand, plays the role of an *accelerating factor*, leading to a deterioration of both epidemiological and macroeconomic outcomes and partly negating the beneficial impacts of the vaccine. A new and improved vaccine in turn can redress the situation. Vaccinations and variants, therefore, can be conceived of as drivers of an intertwined cycle impacting both epidemiological and macroeconomic developments.

## Introduction

Since late 2020, two crucial V-words have changed the dynamics of Covid-19: Vaccines and variants. The introduction of vaccines has raised the hopes of ending the pandemic once and for all but this optimistic belief has been thrown into doubt by the emergence of variants. While it is well known that changes in public health – due, for instance, to a new disease or a new drug – can have remarkable repercussions on economic activity (Pritchett and Summers 2001), health economists generally focus on their *micro-economic* or sectoral effects in the long run, in particular on education provision, productivity, saving and investment (Bloom and Canning 2000, 2008).

In this paper we take a broader perspective and focus on the short to medium run. Our goal is to assess the effects of Covid-19 vaccines and variants *on the macroeconomy*, i.e., on GDP and other aggregate variables over a limited time span, i.e., *at business cycle frequencies* as Covid-19 has, in fact, also had an impact on the amplitude and duration of business fluctuations.

The economy-wide consequences of a change in public health have generally been explored by means of Computable General Equilibrium (CGE) models. Using this approach (Smith et al. 2005) assess the macroeconomic effects of anti-microbial resistance; Keogh-Brown et al. (2010) examine the potential macro economic cost of a modern epidemic; Smith et al. (2009) explore how vaccines affect the macroeconomic impact of influenza in the UK. We adopt a different approach, developing an integrated *agent based macro-epidemiological framework* consisting of a macroeconomic sub-model and an epidemiological network-based compartmental Susceptible, Infectious, Recovered (SIR) sub-model.

Agent based models (ABM) are more granular and more flexible than CGE models. First, by construction ABMs keep track of the behaviour of a large number of interacting agents (households, firms) instead of only a few sectors. Second, the behaviour of each (bounded rational) agent is described by “rules of thumb” which are not necessarily optimal. Finally, market transactions are carried out at prices which are not necessarily market clearing. Market disequilibrium phenomena such as rationing of demand or involuntary accumulation of inventories are pervasive and lead to adaptive adjustment of economic decisions (see Dawid and Delli Gatti (2018)). Aggregate variables such as GDP, consumption etc. are computed “from the bottom up", i.e. summing individual quantities across agents.

We also apply this granular approach to the SIR component of our model. We track contagion along the networks of contacts of each agent both in the workplace and during leisure time. The dynamics of each epidemiological group are therefore micro-simulated instead of being postulated as aggregate laws of motion as in canonical SIR models. With this framework we contribute to a small but growing literature on joint economic and epidemiological dynamics in agent-based settings (e.g. Mellacher 2020; Basurto et al. 2022).

In our macro-epidemiological ABM, the epidemic impacts the labour market (because workers become sick), the market for goods (because households consume less), the healthcare sector (because sick people with serious symptoms must be hospitalized) and public finance (because transfers for sick pay increase while tax revenue declines). These developments negatively affect GDP, causing a slump. Non-pharmaceutical interventions such as a government-mandated lockdown exacerbate the recession in the short run by forcing firms to shut down. The contraction of macroeconomic activity, in turn, feeds back on the epidemiological scenario by reducing the speed of contagion.

From simulations of our ABM we obtain a rich set of results on the effects of vaccination. First of all, as expected, the vaccine significantly contributes to containing contagion and saving lives, even if it is not 100% effective at preventing infection and serious disease. Regardless of the prioritisation strategy, in fact, the cumulative number of infections and of deaths is substantially lower than in the absence of a vaccine.

As to vaccination strategies (priority given to the old vs. priority given to the young and economically active), the literature suggests a potential trade-off between minimizing infections by vaccinating the young first and minimizing fatalities by prioritizing the elderly (Forslid and Herzing 2021; Gollier 2021; Babus et al. 2021; Glover et al. 2022; Brotherhood and Santos 2022). Saad-Roy et al. (2020) find that the impact of vaccines is strongly dependent on the efficacy of the vaccine and the response of the immune system. According to Matrajt et al. (2021), the optimal prioritization strategy depends on vaccine efficacy; in order to minimize deaths, when vaccine efficacy is relatively low, it is optimal to allocate vaccines to the old first. On the contrary, when vaccine efficacy is high, priority should be given to younger age groups.

In terms of the number of infections, the performance of different vaccination strategies does not differ starkly in our simulations: the magnitude of the reduction in the number of cases is broadly similar across different strategies. On the contrary, the ranking of strategies in terms of cumulative fatalities is clear: the vaccination strategy aimed at prioritizing the old – i.e., the agents with higher exposure to the risk of dying – allows to save a remarkably higher number of lives (at the cost of a slightly higher number of infections).

In addition, we find that the vaccine reduces both the frequency and amplitude of the waves of infections and fatalities. The epidemiological effect of vaccination that our simulations reveal, therefore, is a significant *mitigation* of the cyclical dynamics of infections and deaths.

One relevant issue connected to the discussion of vaccination strategies is the vaccination of children; to the extent that infected children play an important role in spreading the disease (see e.g. Gaythorpe et al. (2021) and Silverberg et al. (2022) for reviews of the evidence on the role of children in the SARS-CoV2 pandemic), vaccination may be a sensible approach not only to protect children at risk of serious disease but also to reduce the transmission of the virus from children to adults. While our model does not explicitly consider children as a separate age group, our results suggest that priority should be given to old agents in all contexts. At the same time, however, we also conduct a separate simulation experiment showing that when a significant share of agents remains unvaccinated, epidemiological outcomes deteriorate strongly, suggesting that the maximisation of vaccination rates (potentially including that of children) should be a key policy goal.

As to the macroeconomic effects of vaccination, regardless of prioritisation strategy the vaccine has a significant and persistent positive impact on GDP driven by the increase of consumption that follows from the lower number of infections and fatalities. The decline in infections reduces the perceived risk of contagion, weakens the incentives for social distancing and boosts consumption. The decline of deaths means that the old who survive thanks to the vaccine contribute to consumption while in the absence of vaccination they (and their consumption demand) were “removed” from the economy. We do not find significant differences in macroeconomic outcomes between vaccination strategies, i.e. the prioritisation of young workers does not translate into an aggregate economic gain, relative to prioritization of the old.

We then turn to variants. We assume that variants alter the epidemiological scenario by reducing the effectiveness of the original vaccine in preventing infections and/or serious symptoms and by increasing the transmissibility of the disease. Simulations show that a variant with these features replaces the original virus rapidly and yields a sequence of subsequent waves of contagion. This increase in infections also leads to an increase in deaths, particularly when the variant also reduces the vaccine’s efficacy at preventing serious symptoms (cf. Bernal et al. 2021; Hoffmann et al. 2021; Wall et al. 2021). The economy experiences a sequence of oscillations of GDP which impacts also on government debt as a share of GDP. Variants hence act as an *accelerator*, leading to an increase in the frequency and amplitude of waves of contagion and of fluctuations in macroeconomic activity, partly offsetting the positive effects of the original vaccine.^1^ If the vaccine is adapted to the variant, the amplitude of these waves is mitigated.

The paper is structured as follows. In Section 2 we present a synthetic overview of the model. We provide a detailed description of the model in Appendices A and B. Section 3 presents the epidemiological and macroeconomic dynamics of an epidemic scenario with no vaccine which we use as a benchmark to evaluate the effects of vaccination. The macroeconomic calibration underlying this scenario is discussed in Appendix C. The effects of vaccination are presented in Sections 4, while 5 introduces the emergence and spread of variants. Section 6 discusses the case in which a share of agents remains unvaccinated. Section 7 concludes. Appendix D contains additional simulation experiments and sensitivity analyses. Appendix E contains all parameter values for the macroeconomic and epidemiological sub-models.

## An overview of the model

### The environment

The model is a variant of the ABM presented in Delli Gatti and Reissl (2022). The economy we analyze is populated by households, firms, the banking system and the public sector. The unit of time for the macroeconomic sub-model is a month. The epidemiological sub-model instead runs at the frequency of one week, with every month containing four weeks. In what follows, the subscript *t* indicates a month while the subscript $\tau$ indicates a week.

There are $N_{H}$ households which fall into two categories: $N_{W}$ workers and $N_{F}$ firm owners. For simplicity we assume that only workers can become ill. There are $N_{F}$ firms which fall into three categories: $N_{F}^{k}$ producers of capital goods (K-firms), $N_{F}^{b}$ producers of *basic* (or essential) consumption goods (B-firms) and $N_{F}^l$ producers of non essential or *luxury* consumption goods (L-firms). The set of all consumption goods producers (C-firms) is the union of the sets of B-firms and L-firms, denoted $N^{c}_{F}$. The number of *active* firms may change over time due to entry and exit but never exceeds $N_{F}$. The banking system is represented by a single bank, collectively owned by firm owners.

### The macroeconomic sub-model

In this section we succinctly describe the macroeconomic sub-model. A more detailed description is given in Appendix A.

#### Households

A household indexed with $h\in (1,N_{W})$ is a worker. If alive, workers can be either economically active or inactive. Chiefly for epidemiological purposes, the population of workers is divided into three age groups: young, middle-aged and old. For simplicity agents do not age, i.e. they remain in the age-group to which they are initially assigned. All old agents are assumed to be retired and hence economically inactive. All young and middle-aged agents are initially economically active and constitute the labor force (either employed or unemployed). When an economically active worker falls ill they become economically inactive until their illness ends.^2^

Each economically active worker supplies 1 unit of labor inelastically. If employed, they receive a uniform wage and pay a fraction of this wage to the Government. If unemployed, they receive an unemployment subsidy. Workers who fall ill receive sick-pay. Retired workers receive pensions.

A household indexed with $h=N_{W}+f$ is the owner of the *f*-th firm, $f=1,2,...,N_{F}$. The income of this household consists of dividends, which are equal to a fraction of the after-tax profit of the firm owned by that household. The firm pays out dividends only if profits are positive. Moreover, the firm owners are assumed to jointly own the bank and consequently each receives an equal share of the dividends distributed by the bank. In addition, all households receive interest income on deposits held at the bank, which represent the only financial asset owned by households.

A household’s *consumption budget* is given by a weighted average of past disposable incomes and a fraction of its financial wealth (deposits). The fraction of the consumption budget allocated to B-goods depends on the relative price of B-goods and L-goods. The consumer shops first at B-firms and then at L-firms. The consumer visits two B-firms: the “go-to” supplier and a randomly drawn potential new shopping partner. If the price charged by the former is lower than or equal to that of the latter they will first buy from the go-to supplier and resort to the new seller only if the consumption budget devoted to B-goods is not completely exhausted with the first purchase. Otherwise, they will switch to the new partner (and reverse the order of purchase) with a probability which is increasing with the price set by the go-to partner relative to that of the potential new partner. If the consumer switches to the new partner, the latter becomes their new go-to supplier. The market protocol for L-goods follows the same rules as that for B-goods.

#### Firms

B-firms and L-firms are consumption goods producers (C-firms for short) and follow the same behavioural rules. An active firm indexed with $f\in (1,N^{c}_{F})$ has *market power* and sets its individual price and desired production under uncertainty.

Two *rules of thumb* govern price changes and quantity changes respectively. Excess demand and the relative price $\frac{P_{f,t}}{P_{t}}$ – where $P_t$ is an aggregator of the prices set by C-firms – dictate the *direction* of price adjustment: the firm will increase (reduce) the price next period if it has registered excess demand (supply) and has underpriced (overpriced) the good in the current period. Otherwise it will leave the price unchanged. The *magnitude* of price adjustments is stochastic. Both the direction and the magnitude of quantity adjustment are determined by excess demand. The firm will increase production next period if it has registered excess demand (in the form of a fringe of unsatisfied consumers) in the current period; it will downsize production if it has registered excess supply (i.e., involuntary inventory accumulation).

Technology is represented by a Leontief production function the arguments of which are capital and labor. Both labour and capital productivity are constant, meaning that the model does not feature long-term growth. Once a decision has been taken on desired output, a firm determines how much capital and labor it needs to reach that level of activity. If actual capital is greater than the capital requirement, the rate of capacity utilization will be smaller than one. If actual employment is smaller than the labor requirement, the firm will post vacancies. If the opposite holds true the firm will fire workers. If actual capital is smaller than the capital requirement, the former will be utilized at full capacity but desired output will not be reached and production will be scaled back.

We assume that a C-firm may carry out investment in any given period with a probability $\pi ^{k}<1$. In order to determine its investment demand, the firm calculates a target capital stock based on past utilisation and a target utilisation rate, also taking into account the depreciation of capital and the probability of investing. It invests in capital goods so as to reach this target capital stock, visiting the market for K-goods. The market protocol for this market follows the same rules as those for B-goods and L-goods.

The price adjustment rule for capital goods producers is the same as that of C-firms. The quantity adjustment rule departs from the one adopted by C-firms to take into account the assumption that K-goods are durable and therefore storable. Hence inventories of capital goods carried over from the past can be used to face actual demand. We assume that K-firms are endowed with a linear production function with labor as the only input. Once the price-quantity configuration has been set, a K-firm may post vacancies or fire workers in order to fulfil labor requirements.

Unemployed workers visit a subset of firms chosen at random. Once an unemployed worker finds a firm with an unfilled vacancy a match occurs. The uniform nominal wage is set on the basis of labor market conditions captured by the distance between the current unemployment rate and a threshold unemployment rate. Whenever the unemployment rate is above (below) the threshold the wage will decrease (increase).

#### The banking system

Firms register a financing gap when outlays (to pay for wages and, in the case of C-firms, capital goods) are greater than their available liquidity in the form of accumulated bank deposits. Firms which cannot self-finance their costs demand bank loans.

The bank sets the interest rate on loans and the quantity of credit supplied to firms. The interest rate on loans is set adding a mark up (*external finance premium*) on the risk free interest rate. The external finance premium, in turn, is increasing with the borrower’s leverage. Moreover, the bank determines a maximum amount it is willing to lend to a given borrower, again based on that borrower’s leverage. This means that a firm may be credit rationed and therefore forced to scale down production and/or investment. In every period, borrowers repay a fraction of their outstanding loans.

Households and firms hold deposits at the bank. The interest rate on deposits is a fraction of the fixed risk free interest rate which coincides with the interest rate on Government bonds. If the bank’s profit at the end of a period is positive, it pays a fraction of its after-tax profit as a dividend, which is divided up equally among all firm owners.

#### The public sector

The public sector collects taxes on wage income and profits and provides transfers in the form of unemployment subsidies, sick-pay and pensions, all of which are given by fractions of the current nominal wage. Government expenditure consists of public provision of healthcare services. In case of a public sector deficit, the Government issues bonds. We assume that all issued bonds are purchased by the bank at a fixed interest rate.

#### Demand and supply of healthcare

Government expenditure on healthcare (in real terms) is given by a constant fraction of full employment output, calculated using the initial labor force. We assume that the government uses this amount to spend on the output of both K-firms and C-firms. The goods thus purchased are converted one-for-one into a supply of healthcare.^3^

Even in the absence of an epidemic, a worker may become ill with some probability in any period, but such illness is neither potentially lethal nor infectious to others. As long as an agent is ill, they generate a demand for healthcare which is increasing with their age. If the remaining supply of healthcare in a given period is insufficient to accommodate the agent’s demand, they join a randomised queue to receive treatment. In the case of an epidemic, agents who contract the epidemic disease and develop serious symptoms will also demand healthcare, making it more likely that demand will exceed supply.

#### Entry and exit

The epidemic disease may lead to the death of workers. If a worker dies, their assets are written off, and they may be replaced in each future period by a young worker with a constant probability. During the epidemic the population of living workers can hence temporarily be smaller than $N_W$.

If a firms’ equity becomes negative, it is assumed to go bankrupt and exit.^4^ The exiting firm may then be replaced by a new firm operating in the same sector, with a probability that is increasing with the average profit rate in the sector in question. The new firm receives any fixed capital remaining from the bankrupt firm and receives an injection of liquidity from the owner of latter, who becomes the owner of the new firm. The bank’s equity may become negative due to persistent loan defaults. If this is the case, a bail-in procedure is applied: all firm-owners (who collectively own the bank) make a transfer to the bank until its equity becomes positive.

### The epidemiological sub-model

In this section we briefly describe the epidemiological sub-model. A more detailed outline can be found in Appendix B.

#### The taxonomy of epidemiological groups

The epidemic is characterised by the outbreak of an *infectious* disease which spreads from one subject to the others through *contagion*. At a certain point during the model simulation, a small number of workers are exogenously infected with the epidemic disease and may then spread it to the rest of the population.

Infected agents can be either non-symptomatic or symptomatic. The former are infected agents who do not have symptoms or develop only mild symptoms. In this case the infection can be detected only if the agent is subjected to a random test. Detected non-symptomatic infected agents are quarantined and therefore cannot spread the disease. By assumption, all symptomatic infected agents develop serious symptoms and are detected with certainty. The disease is hence spread only by infected agents who develop mild or no symptoms and remain undetected. For simplicity, we assume that the infected remain contagious for the entire duration of the illness.

The probability to develop serious symptoms is increasing with age. All agents whose infection is detected become inactive and will not have social contacts for the duration of the disease. Only people developing serious symptoms are hospitalized, i.e., they express demand for healthcare services. Non-symptomatic infected agents recover with certainty after a certain number of weeks while those who develop serious symptoms may either recover or die.

#### Contagion

Contagion takes place in three networks: the workplace (employment network), the marketplace (shopping network) and social relations (social network). Each employed worker is linked to all co-workers in the firm for which they work. If a worker is infectious, they can spread the disease to their (susceptible) co-workers.

In addition, all workers are nodes in the shopping network. A certain number of households shop at a given C-firm. If one of these buyers is infectious, they may spread the disease to other households shopping at the same firm. We list all possible connections between the customers of a given firm in a given period and assume that a fixed share of those encounters actually take place (not all customers visit the firm at the same time).

Each worker also has a set of social connections consisting of family and close friends. The total number of social connections is a (very small) fraction of the maximum number of possible undirected connections between workers. While the shopping and employment networks evolve dynamically as agents’ employers and the firms at which they shop change over time, the social network is assumed to be static.

We assume that each agent meets all their connected agents in every week. The set of *potential* new infections is constructed by randomly drawing one infected and one susceptible agent from the set of all connections. We assume that a fraction (the basic transmission rate) of the number of connections in week $\tau$ which involves exactly one infected and one susceptible agent may lead to a new infection. By assumption, the different types of connections have different probabilities of being drawn, being highest for social connections, second highest for workplace connections and lowest for market connections. The basic transmission rate is assumed to be seasonal, being higher during autumn and winter months in the Northern hemisphere (October to April) and lower during late spring and summer (May to September).

Each of these *potential* new infections leads to an actual new infection with a baseline probability equal to 1, but this probability may be reduced if i) one or both agents involved in the respective connection engages in social distancing as described in Section 3 and/or ii) if the susceptible agent is vaccinated as described in Section 4.

#### Recovery, death, re-infection

Infected but non-symptomatic agents recover with certainty, while infected agents with serious symptoms may either recover or die. For any infected individual, the duration of the disease is stochastic, being drawn from a distribution with finite support. Agents with serious symptoms may die in each period in which they are infected, with a probability which increases with age and with excess demand for healthcare. The supply of healthcare services which seriously ill agents actually receive depends on the rate of “capacity utilization" of the healthcare system. In the epidemic scenario, in fact, the demand for healthcare services may rapidly come to exceed the supply.^5^

When the healthcare system becomes overburdened, the demand for healthcare is rationed and an agent who develops serious symptoms may be forced to join a randomised queue. If the seriously ill agent has not died after the duration of the disease, they will recover. We assume that both the effect of age and of excess demand for healthcare on the probability of dying decrease over time until they reach a lower bound as the healthcare system is partly able to adapt to dealing with the novel disease even in the absence of a vaccine (e.g. through the use of existing or new medicines other than vaccines, or simply through increased experience in treating the new disease (Ledford 2020).

Recovered agents who became economically inactive due to the disease will re-enter the labor force as unemployed workers and look for a job. Each recovered agent becomes immune to the epidemic disease (‘natural’ or post-infection immunity) for a number of periods given by a draw from a normal distribution.^6^ Once the drawn number of weeks has passed, the recovered agent becomes susceptible again.

## The baseline epidemic scenario

To construct a baseline scenario against which the effect of a vaccine will be evaluated, we begin by calibrating the macroeconomic sub-model on a situation of **Normal Times**, i.e., in the absence of an epidemic. For this purpose we obtain macroeconomic data for real GDP, consumption, gross fixed capital formation and employment for the Lombardy region of Italy from 1995 to 2017 and follow the same calibration approach as Delli Gatti and Reissl (2022), setting parameter values such that the macroeconomic sub-model replicates a set of moments calculated from these data as closely as possible. The calibration procedure is described in more detail in Appendix C. The resulting parameter values can be found in Table 4 in Appendix E. Starting from the calibrated macroeconomic sub-model, we construct and analyse the baseline **Epidemic scenario** (EP), characterized by spreading of the epidemic disease in the absence of vaccines and variants. In what follows, we denote with $t_{E}$ the *month* and $\tau _{E}$ the *week* in which the epidemic begins.

### Epidemic dynamics

The epidemic is imposed on the model by exogenously changing the status of a small number of agents from *susceptible* to *infected* in $\tau _E$, after which the epidemic disease spreads endogenously as described in Section 2.3 and Appendix B. In the scenario we consider, two features may mitigate the spread of the disease: the adoption of voluntary/spontaneous **social distancing** by private agents, and the implementation of a one-off government-mandated **lockdown**. Government healthcare expenditure remains fixed; Appendix D contains a simulation experiment analysing the results of an increase in healthcare expenditure in the EP scenario.

Social distancing is described by a binary choice model. The probability that an agent will engage in social distancing is increasing in the number of currently infected and *detected* individuals (relative to a fixed threshold value) and the share of other agents who are already distancing, and decreasing in the perceived cost of social distancing. Every time an agent engages in social distancing, three effects occur. Firstly an encounter between a susceptible and an infected individual – which would otherwise lead to an infection with certainty – does so only with probability $1-\beta$ if one of the agents is distancing and $1-2\beta$ if both agents are. Secondly, the number of social and shopping connections decreases linearly up to a lower bound as the share of distancing agents increases. Thirdly, desired consumption of B-goods of an agent engaged in social distancing receives a positive shock, while their desired consumption of L-goods receives a larger negative shock. Social distancing hence leads to both a decrease in overall consumption demand and a change in its composition. The magnitude of both shocks declines over time down to a lower bound while the agent remains in distancing mode. When the agent decides to cease social distancing according to the binary choice model described above, the shocks are removed from their desired consumption. If, at a later point, the agent decides to engage in social distancing once again (because infection numbers have increased again), the shocks to their desired consumption are applied again at their previous (reduced) value. The modelling of social distancing is described in more detail in Appendix B.1.

In addition to voluntary social distancing, there may also be a one-off government mandated lockdown. We assume that the lockdown is imposed when the number of detected weekly new infections reaches an exogenous threshold and remains in force for 12 weeks unless new infections decrease below another threshold prior to this. The lockdown has a range of effects. A fraction of L-firms are closed entirely and cease production for the duration of the lockdown such that no workplace encounters take place at those firms. All firms which remain open move into ‘smart-working’ mode in which only a fraction of workplace encounters take place there. In addition, an upper bound is placed on the number of social and shopping connections which persist during the lockdown and the lockdown lowers agents’ perceived cost of keeping the social distance, making it more likely that an agent will engage in social distancing. Finally, we assume that the lockdown is associated with an increased effort to detect infections. Accordingly, once the lockdown begins, the probability of detecting an infected asymptomatic agent becomes a function of the number of cases detected in the previous week. A more detailed description of the lockdown in the model can be found in Appendix B.2.

Our simulated lockdown is designed to mimic, in a stylised way, the policies implemented by the Italian government starting in March 2020 which, in addition to restrictions of contacts and mobility, also involved the temporary closure of economic activities considered “inessential”, including in manufacturing and non-customer-facing services. A second, ‘softer’ set of regionally differentiated lockdown measures was implemented in late 2020 to combat the second wave (cf. Reissl et al. 2022; Ferraresi et al. 2023). Since the objective of this paper is to examine the impact of vaccines and variants rather than lockdowns, we do not model this second set of lockdown measures. We assume, however, that agents may continue to engage in voluntary social distancing. The EP scenario can hence be considered to mimic the actual institutional setting in Lombardy up to the start of the second wave, and a counterfactual scenario from that point onward. Epidemiological parameters are calibrated such that the model is able to reproduce the actual numbers of infections and deaths observed in that region until the onset of the second wave.

**Fig. 1:**
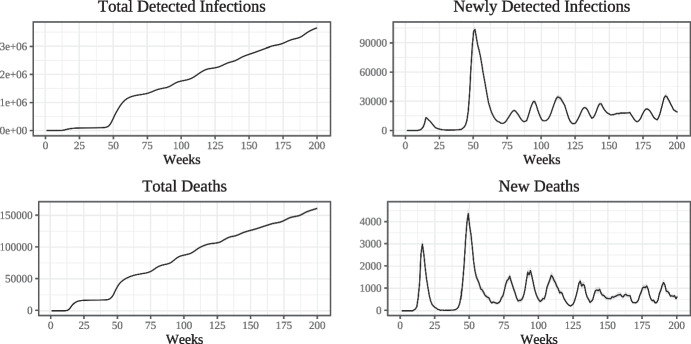
Simulated dynamics of detected infections and deaths (weekly)

In Fig. 1 we show the simulated epidemic curves at weekly frequency. We run the model 100 times with different seeds and compute the mean of the simulated data for each period along with 95% confidence intervals. The top left panel shows the number of cumulative detected infections while the top right panel shows the flow of newly detected infections, with week zero being the beginning of the epidemic $\tau _{E}$. The bottom panels show the same curves for deaths. In all cases, the numbers have been scaled by a factor of $\frac{1}{0.003}$ in order to transform simulated data from our model with a population of 30000 workers into equivalents for Lombardy which has a population of around 10 million.

The adoption of mandated lockdown measures is able to break the first wave of the epidemic at a relatively low level of contagion compared to later waves. Lagged adjustment of social and workplace connections following the lockdown as well as the remaining effects of voluntary social distancing are then able to contain new infections at a low level for some time until gradual relaxation together with the assumed seasonality of the base transmission rate lead to the emergence of a second wave. Since we assume that there is no second lockdown, and since the number of people who have acquired natural immunity through infection is quite low (due to the first lockdown), this second wave is more severe than the first.^7^

**Fig. 2:**
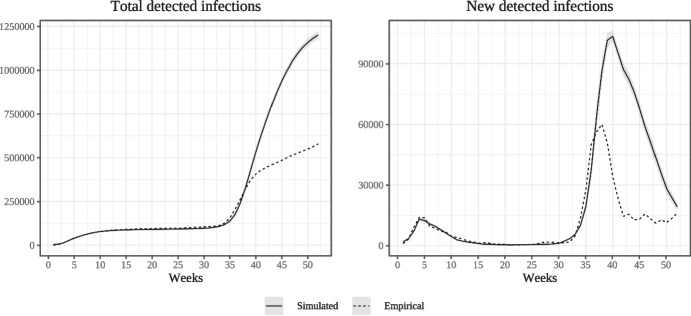
Comparing empirical and simulated infection data (weekly)

Figure 2 compares the empirical dynamics of cumulative detected infections and per-week detected infections in Lombardy to the simulated epidemic curves for the first year of the epidemic, beginning in calendar week 9 of 2020 (the first week of March; denoted as week zero in the diagram). Overall, the model does a good job at reproducing both cumulative and newly detected infections throughout the first wave, although slightly under-estimates the number of infections taking place between the first and second waves. The model also correctly reproduces the timing of the outbreak of the second wave. Once the second wave has started, simulated infections peak at a higher level than in the empirical data as we do not model the second set of lockdown measures.

**Fig. 3:**
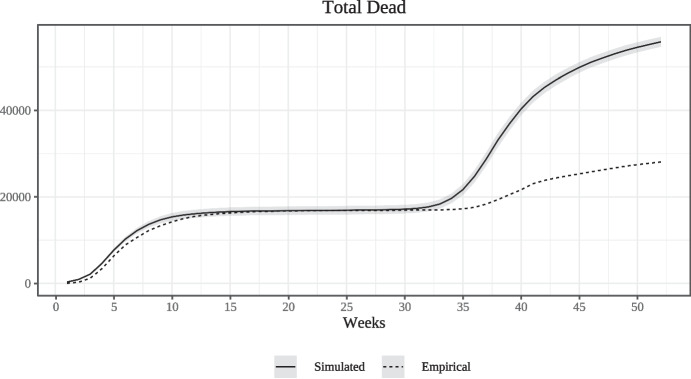
Comparing empirical and simulated deaths (weekly)

Figure 3 compares the simulated and empirical numbers of cumulative deaths for the same time-period as that shown in Fig. 2. The model does a good job at reproducing the cumulative number of deaths at the end of the first wave. During the first and particularly during the second wave, however, simulated deaths increase prior to their empirical counterparts. This is due to the fact that in the model, a patient who develops serious symptoms is as likely to die during the first week in which they are ill as in the last, whereas in the real world, fatalities due to Covid-19 can take place considerable time after the contraction of the disease.

### The macroeconomic effects of the epidemic

**Fig. 4:**
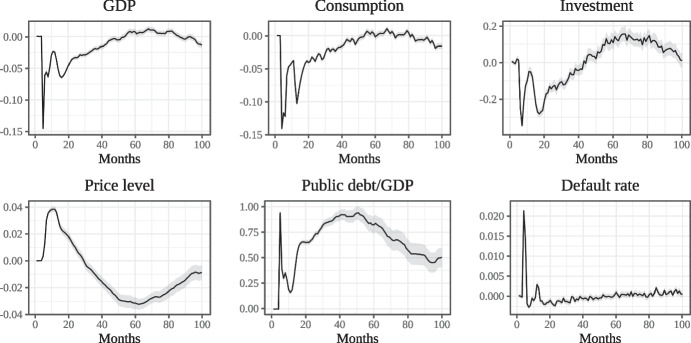
Economic impact of the disease in the Epidemic Scenario (monthly)

In this sub-section, we briefly examine the macroeconomic effects of the epidemic scenario described above. As outlined previously, we run the model 100 times with different seeds. For a given run *r*, for each macroeconomic variable *in levels* – say *x* – we compute the *percent deviation* of the variable in the EP scenario from the scenario of Normal times (NT), i.e. in the absence of an epidemic: $\hat{x}^{r}_{t}=(x^{r,EP}_t-x^{r,NT}_t)/x^{r,NT}_t$. We then compute the mean of these percent deviations across 100 runs, $\hat{x}_{t}$. For variables which are already expressed *in percent terms* (government debt to GDP ratio and the default rate) – say *y* – we compute the *absolute deviation* $\Delta y^{r}_{t}=y^{r, EP}_t-y^{r,NT}_t$ and the mean of the absolute deviations $\Delta y_t$. The time series of these means are plotted in Fig. 4 (along with 95% confidence intervals). Month zero is the first month of the epidemic $t_E$.

By assumption, during the lockdown one third of all L-firms are shut down and cease to produce. This large supply shock is immediately reflected in the aggregate data, leading to a sharp decline in GDP during the lockdown interval. In addition, firms which are forced to close are unable to sell the output they have already produced. Operating costs already incurred are not matched by revenues, which suddenly drop. This liquidity shortfall is the source of the spike in the default rate during the lockdown recession.

In the same interval, there is also a sharp decrease in consumption, partly driven by social distancing and partly by the reduced availability of L-goods. The demand for capital goods falls even more sharply. The reduction in the supply of L-goods – being stronger than the decline in the demand of these goods due to social distancing – leads to an increase in the price of L-goods relative to B-goods and to an increase in the general price level.

At the end of the lockdown, consumption, investment and GDP bounce back but do not regain the levels of Normal Times. The default rate, on the contrary, goes back to normal. The recovery, however, is short lived. As shown above, in the EP scenario the second wave of the epidemic ends up being more severe than the first. Due to the high number of infections during the second wave, there is hence a second large shock to consumption which leads to a renewed decline in GDP, consumption and investment. The second recession begins approximately at month 10 and reaches a trough around month 15.

After the second wave GDP recovers slowly. It takes four years for GDP to reach the level of Normal Times (around month 50). Consumption and investment follow a similar trajectory. GDP, consumption and investment also overshoot the level of NT for some months in the second half of the simulation time span. This overshooting is due to the adaptive rules which characterize agents’ behaviour in the model. Investment in particular overshoots the baseline quite strongly due to firms rebuilding capacity lost during the previous recession. In addition, the rule of thumb which firms use to make their investment decision leads them to over-react to both positive and negative changes in capacity utilisation. Eventually, GDP returns to a level slightly below the baseline, the disease having become endemic.

Initially, the large increase in public outlays due to unemployment benefits and sick pay, coupled with a strong decline in GDP and tax revenue, leads to a sizable increase of the government debt to GDP ratio. The ratio decreases sharply once the lockdown ends but then trends upward again when the second wave begins and only slowly decreases thereafter.

## Vaccination

In this section we address the economic and epidemiological impacts of vaccination in the absence of variants of the virus. We take the EP scenario outlined and analysed above as the baseline against which the effects of vaccination are assessed.

In the vaccination scenario we assume that 11 months after the outbreak of the epidemic (roughly corresponding to the actual start of the vaccination campaign in Italy), a vaccine against the epidemic disease becomes available. Vaccination has two separate effects in our model. Firstly, a vaccinated individual is less susceptible to infection (Vaccine Effectiveness of type 1): the probability that a meeting between an infected and a susceptible individual will result in an infection is reduced by $VE_{1}=0.8$ if the susceptible individual is vaccinated. Secondly, vaccination reduces the vulnerability to serious disease (Vaccine Effectiveness of type 2): if a vaccinated individual is infected, the probability that they develop serious symptoms (which may eventually lead to death) is decreased by $VE_{2}=0.95$. We assume that the vaccine immediately unfolds the above-described effects in a vaccinated individual for a number of weeks (vaccine-induced immunity) drawn from a normal distribution^8^ after which the individual becomes as susceptible to infection and serious disease as they were previously and needs to be vaccinated again.

Vaccination campaigns in the model are continuous (i.e. they also feature re-vaccination) and are characterised by a coverage rate as well as a prioritization rule. We assume that the coverage rate, i.e., the share of the initial population which can be vaccinated in each week, starts at a low level (0.01) and then increases linearly by 0.001 in each week until reaching a level of 0.05. In each week, a number of eligible (susceptible, recovered and undetected infected) individuals corresponding to the coverage rate times the initial population are drawn randomly for vaccination according to probabilities defining the prioritization strategy. In the simulations shown below, we explore three prioritization strategies:

1. Prioritization by age (PA) in which the probability of being drawn increases exponentially with age.
2. Prioritization of workers (PW) in which the probability of being drawn is exponentially higher for economically active agents than for inactive (retired ones).
3. Randomized Vaccination (RV) in which the probability is equal for every agent.

For the moment, we assume that all agents accept to get vaccinated when they receive an offer. The modelling of vaccine effects and vaccination strategies is described in more detail in Appendix B.3. Model simulations show that vaccination reduces both infections and fatalities and therefore the duration and amplitude of the output loss due to the epidemic, and the resulting public sector deficit and debt. Our framework, however, allows to go beyond these intuitive results and gain additional insights about the effects of alternative vaccination strategies both at the macro level – in terms of aggregate health and economic outcomes – and at the meso level, by comparing the number of infections and fatalities between age groups.

**Fig. 5:**
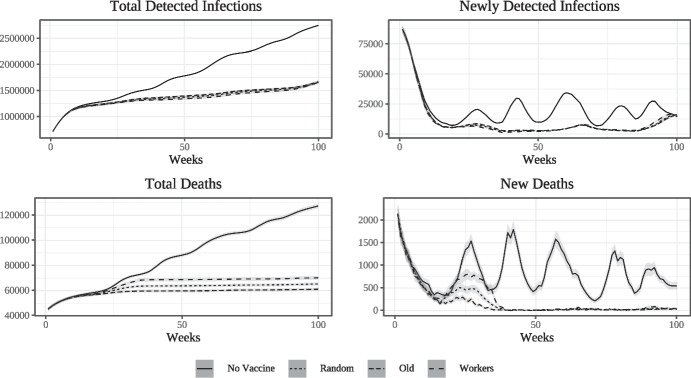
Impact of vaccination on detected infections and deaths for different strategies (weekly)

Figure 5 compares the simulated epidemic curves in the three vaccination cum prioritization scenarios to the baseline given by the Epidemic scenario (EP) presented in Section 3. As in the previous experiments, we run the model 100 times with different random seeds and compute the mean values with $95\%$ confidence intervals for each time period. We consider a time window consisting of 100 weeks starting from the beginning of the vaccination campaign, denoting with $t_{VC}$ the *month* and $\tau _{VC}$ the *week* in which vaccination begins. Period zero in Fig. 5 corresponds to $\tau _{VC}$. The top left panel shows the cumulative number of (detected) infected individuals (computed from the beginning of the epidemic) which occur with and without the vaccine. The top right panel shows the weekly flow of newly detected infections. The bottom panels show cumulative and new deaths respectively. Since vaccination started well after the end of the first wave in Italy, the waves shown in the right panel are the (latter part of the) second wave and subsequent ones.

The vaccine significantly contributes to reducing the number of infections. At the same time, the differences in infection numbers between prioritization strategies are not large; regardless of the strategy, once the campaign begins detected infections quickly diverge in almost identical fashion from those recorded in the absence of vaccination, with the gap between cumulative detected infections with and without the vaccine widening over time. As shown in the top right panel, the vaccine roll-out slightly accelerates the decline of the flow of new infections per week as the second wave dies down. In the absence of a vaccine, there are also multiple subsequent waves, albeit much less pronounced than the second one. With the vaccine, in the time window considered we observe only wavelets of negligible amplitude and frequency. This is because the vaccine-induced immunity is not complete, thus leaving vaccinated agents partially exposed to the risk of infection. In the end *the vaccine acts as a mitigating factor* on epidemiological fluctuations, leading to a reduction of the frequency and amplitude of subsequent waves of contagion.

As expected, the decline in the number of infections coupled with the vaccine’s ability to prevent serious disease also triggers a significant reduction in fatalities, as shown in the bottom panels of Fig. 5. The ranking of prioritisation strategies in decreasing order of number of cumulative fatalities is clear. The best performing strategy along this dimension consists in vaccinating the old first. Interestingly, random vaccination performs better than the strategy based on economic activity. Regardless of the prioritisation strategy adopted, less than one year after the start of the vaccination campaign the number of deceased individuals stabilizes while it continues to grow in the absence of a vaccine. As shown in the bottom right panel, in the presence of vaccination the flow of new deaths rapidly converges closely to zero via dampening oscillations and remains almost flat at zero thereafter while there are multiple additional waves of fatalities in the absence of a vaccine.

In order to assess the difference in the epidemic dynamics across vaccination strategies, Table 1 takes a snapshot of the epidemiological situation after the first year of the vaccination campaign. It shows the total number of deaths and detected infections (averaged across simulations) which occur *during the first year of the vaccination campaign*^9^ under different priority rules, for the whole population and by age groups. First and foremost the table confirms that, independently of the prioritization strategy, vaccination leads to a sizable improvement in the epidemiological situation. Comparing the numbers in the absence of a vaccine with their counterparts under all vaccination strategies, we observe a large reduction in the number of detected cases, and an even more dramatic one in the death toll in all cases.

From the same table we infer that prioritization by age group allows to save more lives (with respect to PW and RV) – in particular among the old – while allowing for a slightly higher number of infections compared to the other vaccination strategies. On the other hand, prioritization by economic activity leads to slightly lower infections – in particular among the young and the middle aged – but higher fatalities. Contrary to old and inactive agents who meet only with social and marketplace connections, employed workers also interact with colleagues. Therefore, a vaccination strategy aimed at prioritizing workers, by protecting individuals with greater connectivity, reduces the overall level of contagion but at the same time leaves the elderly, i.e., the subjects with the highest likelihood of developing serious symptoms, more exposed to the risk of dying.

**Table 1:** Number of deaths and infections in the 1st year, with and without vaccination (average across simulations; extremes of the 95% confidence interval in parentheses)

	No Vaccine	Random	Old	Workers
Total Dead	44633	20713	16657	25653
	(44619, 44648)	(20850, 20577)	(16861, 16453)	(25585, 25721)
Total Detected	1144987	742110	766930	712887
	(1146160, 1143813)	(738130 770442)	(770442, 763418)	(715971, 709802)
Dead (young)	520	243	273	203
	(437, 603)	(190, 296)	(212, 334)	(155, 252)
Dead (middle)	12733	6743	6823	6503
	(12227, 13240)	(6381, 7105)	(6425, 7221)	(6116, 6890)
Dead (old)	31380	13726	9560	18946
	(30573, 32186)	(13241, 14211)	(9068, 10051)	(18273, 19620)

**Fig. 6:**
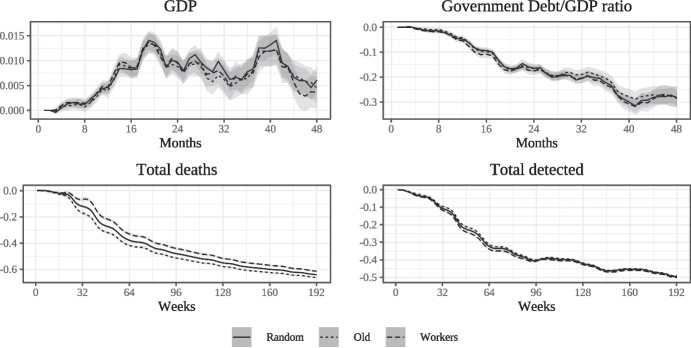
Economic and epidemiological impact of vaccination by strategies (monthly (top) and weekly(bottom))

The macroeconomic effects of vaccination are shown in the upper panels of Fig. 6. We run the model 100 times with different seeds for each vaccination strategy and in every simulation month we calculate the mean deviation from the EP scenario (along with confidence intervals), as described in Section 3, for GDP and the government debt to GDP ratio. These time-series are plotted in the upper panels of Fig. 6, with month zero being the first month of vaccination. To illustrate the relationship with the epidemiological situation, in the lower panels we also report the average % deviations of cumulative deaths and detected infections under vaccination from the EP scenario at weekly frequency for the same time horizon as in the top panels. Week zero is the first week of vaccination.

As already illustrated, the introduction of the vaccine leads to a sizable improvement of the epidemiological situation; cumulative fatalities and detected infections decline steadily relative to the epidemic scenario without vaccine. In parallel, the introduction of the vaccine leads to a considerable improvement of macroeconomic performance, with monthly GDP increasing significantly soon after the introduction of vaccination and eventually settling at a per-period improvement relative to the EP scenario of between 1 and 2 percent. This is essentially due to the positive impact on consumption and aggregate demand resulting from the reduced number of infections and deaths brought about by the diffusion of vaccine-induced immunity. The lower number of infections reduces the prevalence of social distancing and the magnitude of the associated (negative) consumption shock. In addition, the lower number of deaths results in higher aggregate consumption because the old who survive due to the vaccine contribute to aggregate demand while in the EP scenario they (and their consumption demand) were at least temporarily “removed” from the economy. The upper right panel shows a steady and sizable decline of government debt as a fraction of GDP relative to the EP scenario. At the end of the time-window depicted, this ratio is approximately 6% lower than in the epidemic scenario without vaccines. This is essentially due to the boost received by GDP and therefore to the increase of the denominator. Importantly, the figure shows that there are no statistically significant differences in economic outcomes between vaccination strategies, i.e. the prioritisation of economically active agents in vaccination does not appear to translate into an aggregate economic gain, relative to other strategies. In this sense, there is no trade-off between epidemiological and economic outcomes in choosing a vaccination strategy.

## Variants

Vaccination is certainly crucial but may not be sufficient to eradicate a viral epidemic, especially since viruses may mutate rapidly. Mutations that make a virus capable of escaping antibodies survive and spread across the population, becoming “variants of concern” (Zimmer 2021). A sequence of variants of SARS-CoV-2 has emerged around the world over the course of the Covid-19 pandemic. The most important variants of concern for Italy have been Alpha (B.1.1.7) detected in September 2020 in the United Kingdom, Delta (B.1.617) identified in December 2020 in India and Omicron (B.1.1.529) detected in South Africa in November 2021.

In order to examine the effects of virus mutations, we model two variants of the original disease. We assume that both variants feature a sizable increase in the basic transmission rate relative to the original virus and that the seasonality of the transmission rate is less pronounced for the variants.^10^ and that they both reduce the effectiveness of social distancing in preventing infections by $75\%$. The variants differ only in their ability to circumvent the effectiveness of the vaccine against the original virus, which is a centrally important characteristic in determining the ability of a vaccine to become dominant and exacerbate epidemiological dynamics in a setting in which a vaccine is available (cf. Bernal et al. 2021; Hoffmann et al. 2021; Wall et al. 2021). While both of the variants we simulate reduce the vaccine’s effectiveness at preventing infection to the same degree (namely $20\%$), the first variant, $VR_{1}$, leaves the original vaccine’s effectiveness in preventing serious disease unchanged while the second, $VR_{2}$, reduces the latter by $20\%$. We assume in parallel that the vaccine may be adapted over time to be as effective against the variant as the original vaccine was against the original virus, in which case agents need to be re-vaccinated with the new vaccine. Accordingly, we examine four scenarios:

- Variant 1 and original vaccine (VR1-OV)
- Variant 1 and new vaccine (VR1-NV)
- Variant 2 and original vaccine (VR2-OV)
- Variant 2 and new vaccine (VR2-NV)

A variant is introduced in the model by assuming that a few weeks before the start of the original vaccination campaign, a small number of highly connected undetected infected individuals have their infection status exogenously changed from the original virus to the variant, which they then spread among their network of contacts.^11^ Appendix B.4 describes the modelling of the variants in more detail, also illustrating the process of diffusion; in all cases, the variant quickly comes to supplant the original virus, soon accounting for 100% of new infections.

**Fig. 7:**
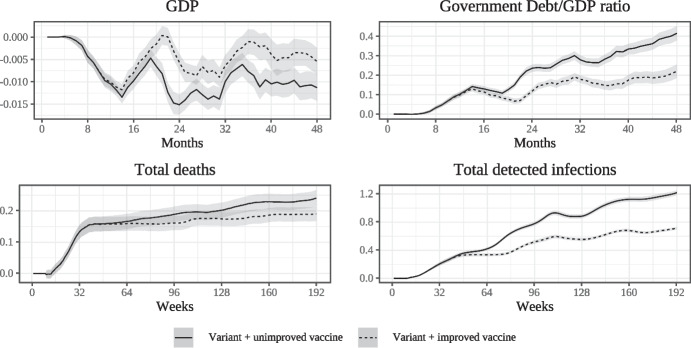
Impact of Variant 1 with and without improved vaccine

Figure 7 summarises the epidemiological and macroeconomic impacts of Variant 1 relative to the epidemic scenario with the original vaccine prioritized according to age (PA). We plot both the VR1-OV and VR1-NV scenarios with deviations from the PA scenario calculated as in Fig. 6. Period 0 is the start of the vaccination campaign.

The emergence of Variant 1 leads to a significant increase in the number of detected infections, particularly in the absence of an improved vaccine. In scenario VR1-NV the new vaccine by assumption becomes available 6 months after the emergence of the variant. Once available, the new vaccine is first administered to any agents who are completely unvaccinated and subsequently to those who had previously received the old vaccine; this new vaccine is partly able to mitigate the increase in infections relative to the PA scenario.

As explained previously, variant 1 by assumption does not reduce the original vaccine’s effectiveness at preventing serious disease. Despite this, the variant leads to a significant increase in fatalities driven purely by the higher number of infections, particularly those occurring in the early stages of the variant’s circulation. As a consequence, the improved vaccine is only partly able to mitigate the increase in fatalities due to variant 1, with the difference between the two scenarios not being statistically significant in this case. The top left panel demonstrates that variant 1 also has a sizable detrimental effect on macroeconomic outcomes. In the absence of an improved vaccine, GDP shows a persistent negative deviation from the PA scenario which oscillates in synchronisation with the magnified waves of infections driven by the variant, with the per-period loss exceeding $2\%$ in some periods. At the same time, the government debt to GDP ratio increases substantially over time. Since the effect on macroeconomic outcomes is chiefly driven by the negative impact of higher infection numbers of consumption, the introduction of an improved vaccine can partly mitigate the deterioration.

**Fig. 8:**
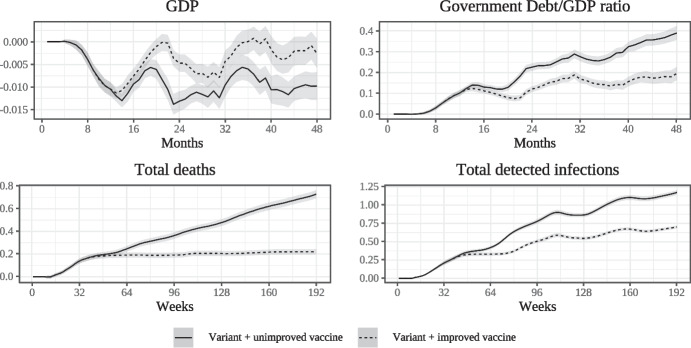
Impact of Variant 2 with and without improved vaccine

Figure 8 shows the scenarios involving Variant 2, which undermines both dimensions of vaccine effectiveness (on the probability of infection and on the probability of serious symptoms). The increase in infection numbers is similar to that shown in Fig. 7 due to the identical characteristics of the variants with regard to transmissibility. By reducing the vaccine’s ability to prevent serious symptoms, however, variant 2 leads to an increase in the death toll which is even more substantial than that caused by variant 1. This can be mitigated only if the healthcare sector is able to swiftly produce and distribute a new and more effective vaccine. Due to the increased number of deaths, the detrimental economic impact of variant 2 is also slightly larger than that of variant 1, with the maximum per-period loss in GDP being slightly higher if no new vaccine becomes available.

Overall, the analysis hence shows that virus variants can act as a strong *accelerating* factor in the epidemiological and macroeconomic dynamics, being able to partly negate the beneficial effects of a vaccine against the original virus. In addition, these variants also lead to considerable macroeconomic losses. The detrimental effects of a variant on both epidemiological and macroeconomic outcomes may however in turn be mitigated if a new and improved vaccine becomes available.

**Fig. 9:**
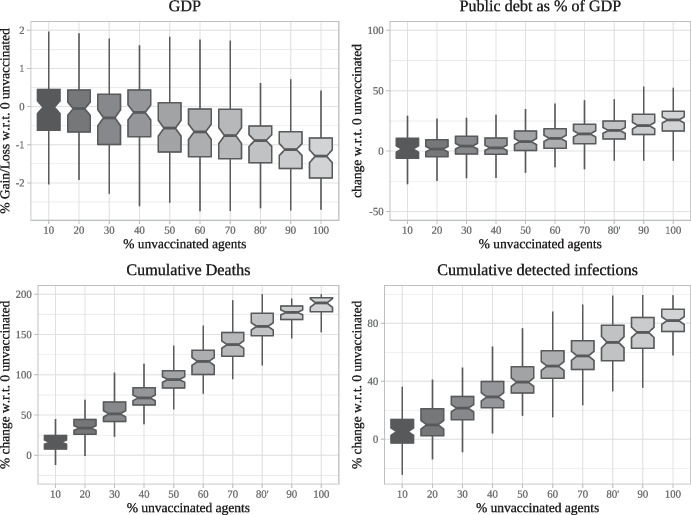
Impact of increasing shares of unvaccinated agents

## The impacts of unvaccinated agents

All scenarios involving vaccines shown up to this point were conducted under the assumption that, once a vaccine becomes available, every agent accepts to be vaccinated as soon as they receive an offer. In the case of the Covid-19 pandemic most countries which have undertaken major vaccine rollouts, however, have experienced difficulty in approaching a vaccination rate anywhere close to 1 even once sufficient doses for everyone had become available. We use our model to examine how simulation results differ when an increasing share of agents remains unvaccinated. In particular, we re-run the scenario of Variant 2 without improved vaccine (VR2-OV) for a total of ten times. In each batch of 100 runs, we increase the share of agents who refuse the vaccine from the baseline of 0 up to 100% in steps of size 10%, with those agents refusing the vaccine being randomly chosen from the population.

The results of the experiments are summarised as boxplots in Fig. 9, with the numbers on the horizontal axes representing the percentage of unvaccinated agents. The top left panel shows the percentage gain/loss in GDP generated over the first 3 years of the vaccination campaign relative to the case of zero unvaccinated agents. The top right panel plots the percentage difference in the public debt to GDP ratio 3 years after the beginning of the vaccination campaign relative to the case of zero vaccinated agents. The bottom panels show the percentage changes in cumulative deaths and infections relative to the case of zero unvaccinated agents 3 years after the beginning of the campaign.

As one might expect, an increasing share of unvaccinated agents has a rather drastic impact on epidemiological outcomes, with cumulative detected infections but especially cumulative deaths three years into the vaccination campaign increasing very strongly with the share of unvaccinated agents. The presence of unvaccinated agents however also has a sizable negative effect on economic outcomes, leading to significantly lower values of GDP and large increases in the public debt to GDP ratio especially when the share of unvaccinated agents is high.

## Conclusion

Using macro-epidemiological agent-based model calibrated on the Lombardy region of Italy, this paper explored the epidemiological and macroeconomic effects of vaccines and virus variants in the context of the Covid-19 pandemic.

As expected, the availability of a vaccine strongly slows down the pace of the epidemic in our model and in particular is able to save a large number of lives, acting as a significant *mitigating factor* of the cyclical dynamics of infections and deaths. At the same time, the lower numbers of infections and fatalities under vaccination also translate into a substantial economic gain in the form of a positive impact on GDP. Different vaccination strategies do not differ greatly in terms of epidemiological and macroeconomic results, but a strategy prioritizing old agents for vaccination emerges as the best choice for minimising the number of deaths. Importantly, the choice of vaccination strategy in our model does not imply an economic trade-off, in that a prioritization of economically active agents does not lead to significantly better economic outcomes than other strategies.

The emergence of a variant of the original virus, by contrast, plays the role of an *accelerating factor*, counteracting the mitigating effects of the vaccine on epidemiological dynamics and also leading to a substantial economic loss. These adverse developments can in turn be addressed by the introduction of a new and improved vaccine. The emergence and diffusion of new and more transmissible variants counteracted by new and improved vaccines may come to represent the new normal in the future.

Finally, the presence of a share of agents refusing the vaccine predictably leads to a sizable deterioration in epidemiological outcomes, but the model in addition shows that the associated economic losses may also be quite substantial.

## Figures and Tables

**Fig. 10:**
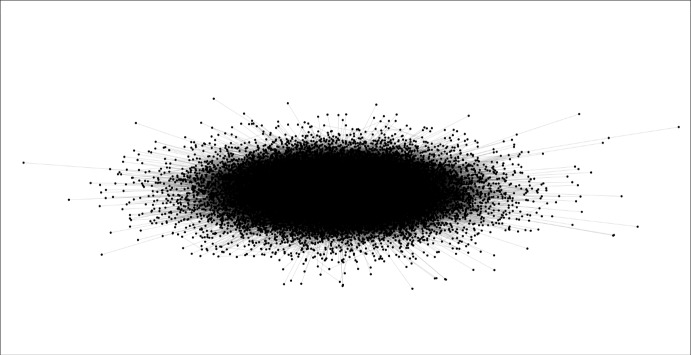
Network of agents during normal times

**Fig. 11:**
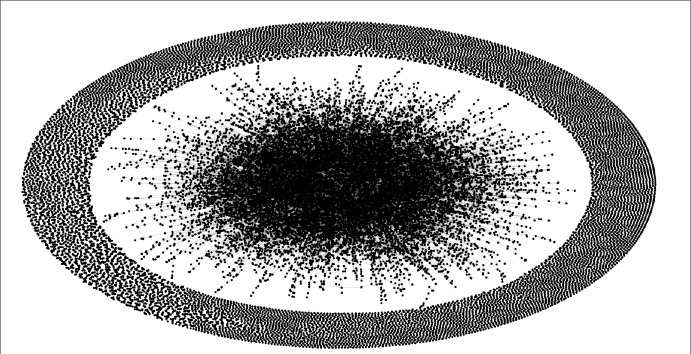
Network of agents under lockdown

**Fig. 12:**
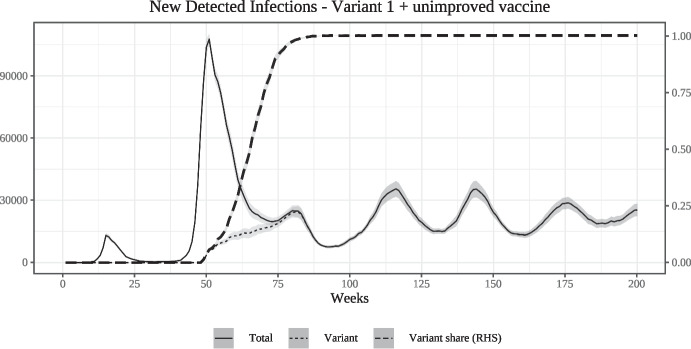
Spreading of Variant 1

**Fig. 13:**
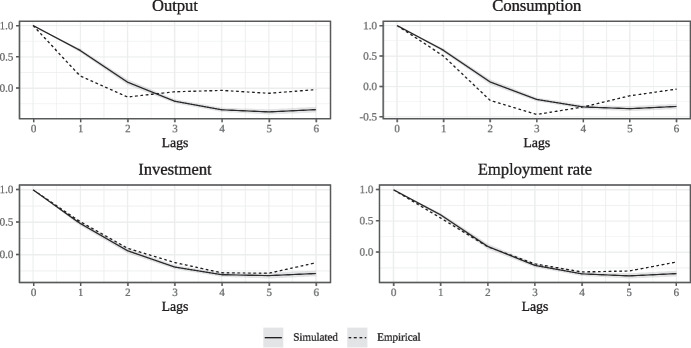
Empirical and simulated autocorrelations

**Fig. 14:**
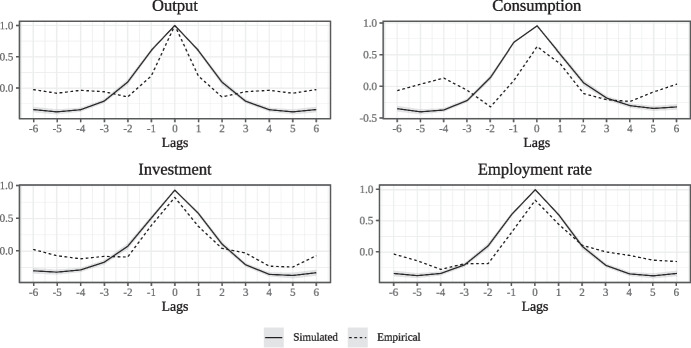
Empirical and simulated cross-correlations

**Fig. 15:**
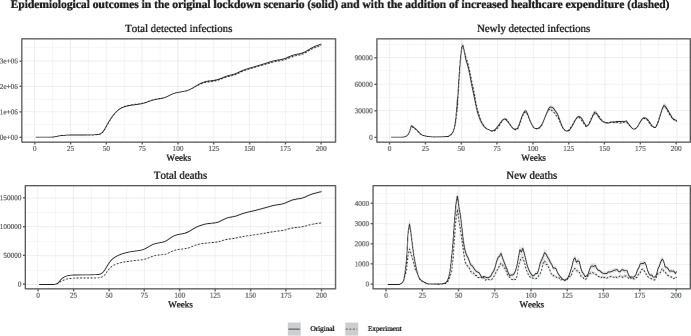
Epidemiological effects of increased healthcare expenditure

**Fig. 16:**
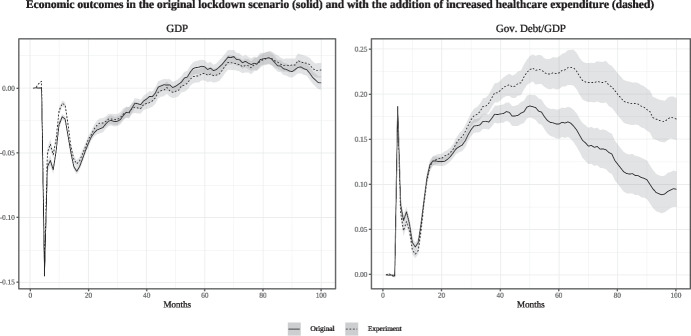
Economic effects of increased healthcare expenditure

**Fig. 17:**
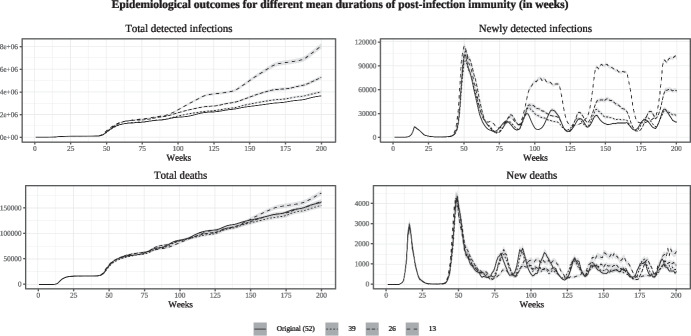
Epidemiological effects of varying the mean duration of post-infection immunity

**Fig. 18:**
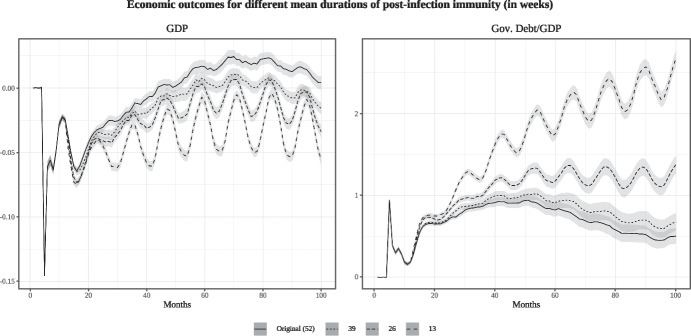
Economic effects of varying the mean duration of post-infection immunity

**Fig. 19:**
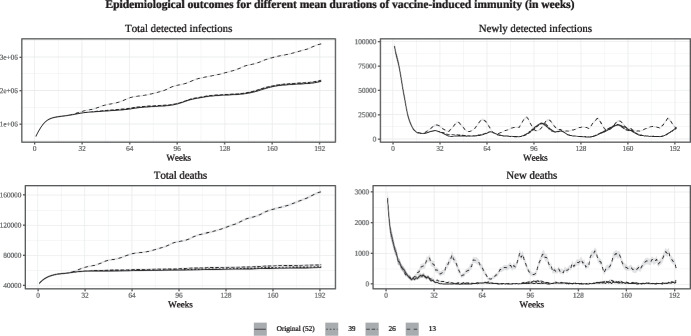
Epidemiological effects of varying the mean duration of vaccine-induced immunity

**Fig. 20:**
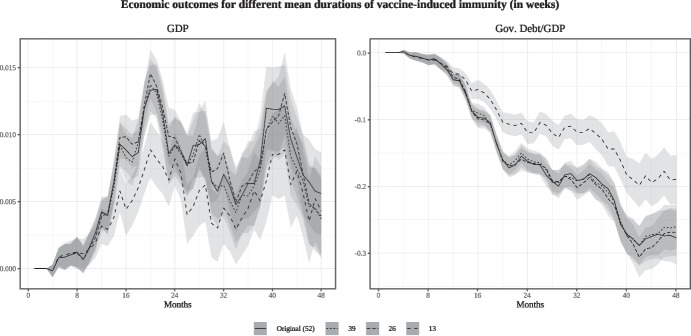
Economic effects of varying the mean duration of vaccine-induced immunity

## Appendix A: The macroeconomic sub-model

In this appendix we provide a detailed description of the macroeconomic sub-model. The values of all economic model parameters introduced below are given in Appendix E.

### A.1 Households

The economy is populated by $N_{H}$ households, of which $N_{W}$ are workers and $N_{F}$ are firm owners. Households are indexed with $h=1,2...N_W, N_W+1,...,N_H$. Households indexed with $h\in (1,N_W)$ are workers; households indexed with $h\in (N_W+1,N_H)$ are firm owners. Of course, the cardinality of the set of firm owners is $N_F=N_H-N_W$. As there is one owner household per firm, it coincides with the cardinality of the set of firms.

#### A.1.1 Workers

Workers can be economically active (employed or unemployed) or inactive (sick or retired). Each active worker supplies 1 unit of labor inelastically. If employed, they receive the uniform nominal wage $w_t$ and pay a fraction $t_w$ (the tax rate on wages) of this wage to the Government.

If unemployed, the worker searches for a job visiting a subset $z_e$ of firms chosen at random. Once an unemployed worker finds a firm with an unfilled vacancy they stop searching and the match occurs. Unemployed workers who have not succeeded in finding a job receive an unemployment subsidy from the Government equal to a fraction of the wage: $s_u w_t$. A sick worker receives sick-pay $s_s w_t$ from the Government. Each retired worker receives a state pension $s_p w_t$. The parameters $s_u, s_s, s_p$ are the *replacement rates* in the case of unemployment subsidy, sick-pay and pension.

#### A.1.2 Firm owners

The household indexed with $h=N_W+f$ is the owner of the *f*-th firm, $f=1,2,...,N_F$. The income of this household consists of dividends, which, in turn, are equal to a fraction $\omega$ (the pay-out ratio) of the after-tax profit $(1-t_{\Pi })\Pi _{f,t-1}$ where $t_{\Pi }$ is the tax rate on profit and $\Pi _{f,t-1}$ is profit generated in the previous period. The firm pays out dividends only if $\Pi _{f,t-1}>0$. If a firm faces a loss, its net worth will go down correspondingly and the firm will not distribute dividends. Moreover, the firm owners are assumed to jointly own the representative bank and consequently each firm owner receives an equal share of the dividends distributed by the bank: $\omega (1-t_{\Pi }) \Pi _{b,t-1}$.

#### A.1.3 Households as consumers

Households receive income and interest payments $r_d D_{h,t-1}$ where $r_d$ is the interest rate on deposits and $D_{h}$ are deposits. Hence the disposable income of household $h\in (1,N_W)$ is:

A.1 $$\begin{aligned} Y_{h,t}= {\left\{ \begin{array}{ll} (1-t_w)w_t +r_d D_{h,t-1}&{} \text {if h is employed},\\ s_u w_t +r_d D_{h,t-1}&{} \text {if h is unemployed},\\ s_s w_t +r_d D_{h,t-1}&{} \text {if h is sick (but not retired)},\\ s_p w_t +r_d D_{h,t-1}&{} \text {if h is retired}\\ \end{array}\right. } \end{aligned}$$

while the disposable income of household $h\in (N_W+1,N_H)$ is

A.2 $$\begin{aligned} Y_{h,t}=(1-t_{\Pi })\omega \left( {1_{\pi }^f}\Pi _{f,t-1}+\frac{1}{N_F} {1_{\pi }^b}\Pi _{b,t-1}\right) +r_d D_{h,t-1}, \end{aligned}$$

where *f* is the firm owned by household *h* and ${1_{\pi }^f}$ is an indicator function taking the value 1 if the profit of *f* is positive and 0 otherwise. ${1_{\pi }^b}$ is the same indicator function for the bank.

A household’s consumption decision proceeds in four steps. First, the household constructs a proxy of permanent income $\overline{Y}_{h,t}$ using an adaptive algorithm: $\overline{Y}_{h,t}=\xi _Y \overline{Y}_{h,t-1}+(1-\xi _Y)Y_{h,t}$ where $\xi _Y\in (0,1)$ is a memory parameter. $\overline{Y}_{h,t}$ is hence a weighted average of past disposable incomes with exponentially decaying weights.

Second, the household determines the desired *consumption budget*:

A.3 $$\begin{aligned} C_{h,t}=\overline{Y}_{h,t}+c_W D_{h,t-1} \end{aligned}$$

where $c_W\in (0,1)$ is the propensity to consume out of financial wealth, which, in this setting, coincides with deposits.

Third, the consumer allocates $c^b \in (0,1)$ of $C_{h,t}$ to the consumption of basic/essential goods (B-goods hereafter). Therefore $(1-c^b)$ of $C_{h,t}$ will be devoted to purchasing luxury/inessential goods (L-goods).^12^ We assume that $c^b$ is a decreasing function of the (average) price of B-goods relative to L-goods.^13^

Fourth, the consumer goes to the market to purchase consumption goods which can be either B-goods or L-goods. Consider first the market for B-goods. B-firms are indexed with $f\in (1,N^b_F)$ where $N^b_F$ is the cardinality of the set of B-firms.

In period t, the consumer visits two firms in this set: a “go-to” supplier and a potential new partner, the latter being randomly drawn from the population of B-firms (excluding the go-to supplier). The consumer then compares the prices. If the price charged by the go-to firm (say $$P_{0}$$) is lower than or equal to that of the potential new partner ($$P_{1})$$, they will stick to the former and will shop at the latter only if the consumption budget is not completely exhausted with the first purchase. Otherwise, they will switch to the new partner (and reverse the order of purchase) with a probability $$\pi ^c$$ which is increasing in the price set by the go-to partner relative to that of the potential new partner: $$R^b_{0,1}=P_{0}/P_{1}$$. In symbols:

A.4 $$\begin{aligned} \pi ^c= {\left\{ \begin{array}{ll} 1-\exp \gamma _p ({1-R^b_{0,1}})&{} \text {if } R^b_{0,1}>1,\\ 0&{}\text {if } R^b_{0,1}\le 1\\ \end{array}\right. } \end{aligned}$$

where $\gamma _p>0$. If the consumer actually switches to the new partner, the latter becomes their new go-to partner in period t+1.

This *partner selection* mechanism implies an implicit *negative elasticity* of the demand for the good produced by the *f*-th firm with respect to the price it charges relative to that of its competitors. Consider firm *f*, $f\in (1,N^b_F)$. The higher is $P_f$ relative to $P^b_{t}$, the higher the probability that the customers of the *f*-th B-firm will switch to a new partner, reducing the demand for the *f*-th B-good accordingly.^14^ If a firm goes bankrupt, all the households who have this firm as their go-to supplier will randomly choose a different go-to supplier.

If, at the end of their visits to B-firms, the household has not spent the consumption budget allocated to B-goods, they will save involuntarily. This market protocol does not guarantee equilibrium. Queues of unsatisfied consumers (involuntary savers) at some firms may coexist with involuntary inventories of unsold goods at some other firms.

The market protocol for L-goods follows the same lines as that of B-goods. L-firms are indexed with $f\in (N^b_F+1,N^c_F)$ where $N_F^c=N_F^b+N_F^l$ is the cardinality of the set of C-firms (the union of B-firms and L-firms). The consumer has one “go-to” L-supplier (who sets the price $P_0$) and one potential partner ($P_1$). They will stick to the former and shop at the latter only if the budget allocated to L-goods is not completely exhausted with the first purchase in the case in which $R^l_{0,1}=\frac{P_{0}}{P_{1}} \le 1$. They will switch to the new partner (and reverse the order of purchase) with probability $\pi ^c=1-\exp \gamma _p ({1-R^l_{0,1}})$ if $R^l_{0,1}>1$. If the budget allocated to L-goods has not been entirely spent, the household will add the residual to their savings.

Total saving is equal to the sum of voluntary or desired saving (i.e., the difference between disposable income and the budget allocated to consumption) and involuntary saving. This is tantamount to saying that actual saving is equal to the difference between current income and actual consumption of B-goods and L-goods. Savings are used to accumulate financial wealth in the form of bank deposits.

### A.2 Firms

There are $$N_F$$ firms, of which $$N^b_F$$ produce B-goods, $$N^l_F$$ produce L-goods and $$N^k_F$$ produce capital goods (K-goods). Firms are indexed with $$f=1,2,...N^b_F,N^b_F+1,...N^c_F, N^c_F+1,....N_F$$ where $$N^c_F=N^b_F+N^l_F $$. In words: firms indexed with $$f\in (1,N^b_F)$$ produce B-goods; firms indexed with $$f\in (N^b_F+1,N^c_F)$$ produce L-goods; firms indexed with $$f\in (N^c_F+1,N_F)$$ produce K-goods.

#### A.2.1 C-Firms

B-firms and L-firms are consumption goods producers (C-firms for short) and follow the same behavioural rules. Here we describe the behaviour of a generic C-firm.

The firm has *market power* and sets its individual price and desired production under uncertainty. It knows from experience that if it charges a higher price it will receive smaller demand but it does not know the actual demand schedule (i.e., how much the consumers would buy at any given price). Hence the firm is unable to maximize profit since marginal revenue is unknown. We assume that the firm charges a price as close as possible to the average price and sets a quantity as close as possible to (expected) demand.

The *f*-th firm, $f \in (1:N^c_F)$, must choose in *t* the price and desired output for $t+1$, i.e., the pair $\left( P_{f,t+1},Y_{f,t+1}^{*}\right)$. Desired output is determined by expected demand $Y_{f,t+1}^{*}=Y_{f,t+1}^{e}$. The firm’s information set in *t* consists of (i) the relative price $\frac{P_{f,t}}{P_{t}}$ – where $P_{f,t}$ is the price of the f-th good and $P_{t}$ is the average price level – and (ii) excess demand

A.5 $$\begin{aligned} \Delta _{f,t}:=Y^d_{f,t}-Y_{f,t} \end{aligned}$$

where $Y^d_{f,t}$ is the demand for the f-th good and $Y_{f,t}$ is actual output. $\Delta _{f,t}$ shows up as a queue of unsatisfied customers if positive and as an inventory of unsold goods if negative. By assumption C-goods are not storable. Therefore involuntary inventories cannot be employed to satisfy future demand.

The firm makes use of two *rules of thumb* which govern price changes and quantity changes respectively.

The *price adjustment* rule is:

A.6 $$\begin{aligned} P_{f,t+1} = {\left\{ \begin{array}{ll} P_{f,t} (1+ \mathbf {1_u}\rho _p ) &{}\text {if } \Delta _{f,t} >0\\ P_{f,t} (1- \mathbf {1_o}\rho _p) &{}\text {if } \Delta _{f,t} \le 0 \end{array}\right. } \end{aligned}$$

where $\rho _p$ is a random positive number, $\rho _p \sim \mathcal {U}(0,\overline{\rho _p})$. $\mathbf {1_u}$ is an indicator function which takes value equal to 1 if the firm has underpriced the good (i.e., if $\frac{P_{f,t}}{P_{t}}<1$), 0 otherwise. Analogously $\mathbf {1_o}$ is equal to 1 if the firm has overpriced (i.e., if $\frac{P_{f,t}}{P_{t}}>1$), 0 otherwise.

Excess demand $\Delta _{f,t}$ and the relative price $\frac{P_{f,t}}{P_{t}}$ dictate the *direction* of price adjustment: the firm will increase (reduce) the price next period if it has registered excess demand (supply) and has underpriced (overpriced) the good in the current period. The *magnitude* of the adjustment is stochastic. The upper bound of the support of $\rho _p$ limits the admissible price change. We also assume that the firm will never set a price lower than its average cost.

Since the quantity to be produced is equal to expected demand, the *quantity adjustment* rule takes the form of an updating rule for expected demand:

A.7 $$\begin{aligned} Y^*_{f,t+1}= Y^e_{f,t+1}=Y_{f,t} +\rho _q \Delta _{f,t} \end{aligned}$$

where $\rho _q \in (0,1)$. Both the direction and the magnitude of quantity adjustment are hence determined by excess demand.

Technology is represented by a Leontief production function, giving the maximum output the firm can produce in *t*: $\widehat{Y}_{f,t} = \min (\alpha _N N_{f,t}, \alpha _K K_{f,t})$ where $\alpha _N$ and $\alpha _K$ represent labor and capital productivity respectively, which are assumed constant. Once a decision has been taken on desired output in $t+1$, the firm retrieves from the production function how much capital it needs in $t+1$ to reach that level of activity (capital requirement): $K^*_{f,t+1}=Y^*_{f,t+1}/\alpha _K$. If actual capital $K_{f,t+1}$ is greater than the capital requirement, the *rate of capacity utilization* $x_{f,t+1}=\frac{K^*_{f,t+1}}{K_{f,t+1}}$ will be smaller than one. The labor requirement will be: $N^*_{f,t+1}=\frac{\alpha _K}{\alpha _N}K^*_{f,t+1}$. If actual employment in *t*, $N_{f,t}$, is smaller than the labor requirement in $t+1$, the firm will post vacancies. If the opposite holds true the firm will fire workers in random order. In this scenario, provided it is able to hire any additional required labor, the firm can reach the desired level of production.

On the other hand, if actual capital $K_{f,t+1}$ is smaller than the capital requirement, the former will be utilized at full capacity (the rate of capacity utilization will be $x_{f,t+1}=1$). The inputs being perfect complements, employment will be proportional to the available capital: $N_{f,t+1}=\frac{\alpha _K}{\alpha _N}K_{f,t+1}$. Hence desired output will not be reached: $Y_{f,t+1}=\alpha _K K_{f,t+1}<Y^*_{f,t+1}$.

Given a stock of undepreciated capital, actual capital in $t+1$ $K_{f,t+1}$ will be determined by investment carried out in *t*, $I_{f,t}$. By assumption, in planning investment, the firm sets a *benchmark* equal to the capital stock used in production “on average” since the beginning of activity $\overline{K}_{f,t}$. This, in turn, is computed by means of an adaptive algorithm, i.e., the weighted average of past utilized capital from the beginning of activity until *t* with exponentially decreasing weights. In computing this weighted average, the firm employs a memory parameter $\xi _K \in (0,1)$. Capital depreciates at the rate $\delta$. Moreover we assume that C-firms may invest in each period with a probability $\pi ^k$. Hence investment necessary “on average” to replace worn out capital is $\frac{\delta }{\pi ^k}\overline{K}_{f,t+1}$.

We assume, moreover, that the firm plans to maintain, in the long run, a capital stock buffer. Therefore the *target* capital stock is equal to $K^{T}_{f,t+1}=\frac{1}{\bar{x}}\overline{K}_{f,t}$ where $\bar{x} \in (0,1)$ is the desired long run capacity utilization rate. Net investment is $K^{T}_{f,t+1}-K_{f,t}$. Therefore gross investment in *t* is:

A.8 $$\begin{aligned} I_{f,t}=\left( \frac{1}{\bar{x}}+\frac{\delta }{\pi ^k} \right) \overline{K}_{f,t}-K_{f,t} \end{aligned}$$

Once investment has been determined, C-firms go to the market for K-goods. The protocol for this market follows the same lines as those of B-goods and L-goods. The *f*-th C-firm, with $f\in (1,N^c_F)$, has one go-to K-supplier (which sets the price $P_0$) and one potential new partner (which sets the price $P_1$) in the population of K-firms, indexed with $f\in (N^c_F+1,N_F)$. If $R^k_{0,1}=\frac{P_{0}}{P_{1}} \le 1$ the C-firm will stick to the go-to supplier and shop at the new partner only if the investment budget $I_{f,t}$ is not completely exhausted with the first purchase. It will switch to the new partner (and reverse the order of purchase) with probability $\pi ^c=1-\exp \gamma _p ({1-R^k_{0,1}})$ if $R^k_{0,1}>1$. If the C-firm’s demand for K-goods has not been completely satisfied, it is forced to “save” the unspent portion of the investment budget. Therefore actual investment may turn out to be lower than planned investment.

The uniform nominal wage is set on the basis of labor market conditions captured by the distance between the current unemployment rate $u_{t}$ and a threshold $u^T$. Whenever the unemployment rate is above (below) the threshold the wage will decrease (increase). The wage updating mechanism therefore is:

A.9 $$\begin{aligned} w_{t+1}= {\left\{ \begin{array}{ll} w_{t} \left[ 1+u_{up}\left( u^T-u_{t} \right) \right] ; &{} u^T-u_t > 0 \\ w_{t} \left[ 1+u_{down}\left( u^T-u_{t} \right) \right] &{} u^T-u_t < 0 \\ \end{array}\right. } \end{aligned}$$

where $u_{up}$ and $u_{down}$ are positive parameters. We will assume that $u_{up}> u_{down}$ to capture the downward stickiness of nominal wages.

#### A.2.2 K-firms

Firms indexed with $f\in (N^c_F+1,N_F)$ are capital goods producers. The price adjustment rule followed by the f-th K-firm is Eq. A.6 but the indicator functions should be re-interpreted. Denoting with $P_{f,t}$ the individual price and with $P^k_{t}$ the average price of capital goods, the function $\mathbf {1_o}$ is equal to 1 if the K-firm in question has overpriced the good (i.e., if $\frac{P_{f,t}}{P^k_{t}}>1$), 0 otherwise. Analogously, $\mathbf {1_u}$ takes value equal to 1 if the K-firm has underpriced the good (i.e., if $\frac{P_{f,t}}{P^k_{t}}<1$), 0 otherwise.

The quantity adjustment rule departs from the one adopted by C- firms (see Eq. A.7) to take into account the assumption that K-goods are durable and therefore storable: inventories of capital goods can be carried on from one period to another, depreciating at a rate $\delta ^k$ in each period. The quantity adjustment rule of the *f*-th K-firm is:

A.10 $$\begin{aligned} Y^*_{f,t+1}=Y_{f,t} +\rho _q \Delta _{f,t}-Y^k_{f,t} \end{aligned}$$

where $Y^*_{f,t+1}$ is the desired scale of activity, $Y_{f,t} +\rho _q \Delta _{f,t}=Y^e_{f,t+1}$ is expected demand, $Y^k_{f,t}$ is the inventory of firm *f* and $\Delta _{f,t}$ is excess demand for the K-good produced by firm *f* at time *t*. K-firms are endowed with a linear production function with labor as the only input.

### A.3 The banking system

Once the quantities to be produced as well as desired investment have been set and the cost of inputs determined, firms have to deal with financing. Consider a generic firm *f*. If the firm’s internal liquidity (i.e., the current deposits held at the bank) $D_{f,t}$ are greater than the costs to be incurred, the firm can finance production and investment (if any) internally. If, on the other hand, liquidity is not sufficient to carry out production and investment up to the desired level, the firm applies for a loan to fill its financing gap which is given by

A.11 $$\begin{aligned} F_{f,t}= w N_{f,t} + \mathbf {1_c} P^k _{t-1} I_{f,t} - D_{f,t} \end{aligned}$$

where $\mathbf {1_c}$ is an indicator function which assigns value 1 to C-firms and 0 to K-firms (since only C-firms purchase capital goods). We assume that C-firms assess the financing gap (and the demand for loans) before accessing the market for capital goods. Hence capital goods to be bought in *t* are priced with the “average” price of capital goods $P^k _{t-1}$.

For simplicity we assume there is only one bank which collects deposits from firms and households, supplies credit to firms and purchases government bonds. The bank decides (i) the interest rate to be charged to each borrower and (ii) the size of the loan (which may be different from the borrower’s financing gap). Both decisions are affected by the borrower’s leverage $\lambda _{f,t}$:

A.12 $$\begin{aligned} \lambda _{f,t} = \frac{L_{f,t}}{E_{f,t}+L_{f,t}} \end{aligned}$$

where $L_{f,t}$ is the firm’s debt and $E_{f,t}$ is net worth.

The bank makes an assessment of the probability of default, which is increasing with leverage. The perceived probability of default for the *f*-th C-firm, $f\in (1,N^c_F)$, is:

A.13 $$\begin{aligned} \pi ^b_{f,t} = \frac{e^{b_{0c} + b_{1c} \lambda _{f,t}}}{1+e^{b_{0c} + b_{1c} \lambda _{f,t}}} \end{aligned}$$

with $b_{0c}<0$ and $b_{1c}>0$. Analogously, the assessed probability of default for the f-th K-firm, $f\in (N^c_F+1,N_F)$, is:

A.14 $$\begin{aligned} \pi ^b_{f,t}= \frac{e^{b_{0k} + b_{1k} \lambda _{f,t}}}{1+e^{b_{0k} + b_{1k} \lambda _{f,t}}} \end{aligned}$$

with $b_{0k}<0$ and $b_{1k}>0$. The interest rate charged by the bank to each firm is determined by adding an *external finance premium* (Bernanke et al. 1996) to the exogenous risk free interest rate *r*. The external finance premium is increasing with the probability of default which in turn is (non-linearly) increasing with leverage. The interest rate charged to the generic *f*-th firm is:

A.15 $$\begin{aligned} r_{f,t}=\mu f(r, \lambda _{f,t}) \end{aligned}$$

where the function *f*(.) is increasing in both arguments.^15^

In order to determine the size of the loan given to a firm *f*, the bank first sets a tolerance level for the potential loss $\Gamma _{b,t}$ on credit extended to any individual borrower as a fraction $\phi _b$ of its net worth: $\Gamma _{b,t} =\phi _b E_{b,t}$. The borrower’s total debt in *t* will be $\Phi _{f,t}+L_{f,t-1}$ where $\Phi _{f,t}$ is the new credit line to be supplied in *t*. We assume the bank sets the new credit line in order to equate the expected loss on loans extended to the *f*-th firm to the tolerance level: $(\Phi _{f,t}+L_{f,t-1})\pi ^b_{f,t}=\phi _b E_{b,t}$. Therefore the new credit line is:

A.16 $$\begin{aligned} \Phi _{f,t} = \frac{\phi _b}{\pi ^b_{f,t}}E_{b,t}-L_{f,t-1} \end{aligned}$$

Given the current exposure of the bank to the firm *f*, the new credit line is increasing with the bank’s net worth and decreasing with the firm’s leverage. The size of the loan actually granted to firm *f* at time *t* will be the minimum between the new credit line and the financing gap:

A.17 $$\begin{aligned} \dot{L}_{f,t} = \min (\Phi _{f,t}, F_{f,t}) \end{aligned}$$

If the latter is greater than the former the firm will be rationed on the credit market and therefore forced to scale down its investment and/or production. In addition to making interest payments, firms in each period repay a fraction $\zeta$ of their total debt to the bank. The bank remunerates deposits and earns interests on loans and on Government bonds. The interest rate on deposits is determined by marking down the risk-free interest rate.

### A.4 Net worth updating

In every period, each firm’s net worth $E_{f}$ is updated by means of retained net profits:

A.18 $$\begin{aligned} E_{f,t+1} =E_{f,t}+(1-t_{\Pi })(1-\omega )\Pi _{f,t} \end{aligned}$$

Also the bank’s net worth is updated by means of retained profits:

A.19 $$\begin{aligned} E_{b,t+1} = E_{b,t} + (1-t_{\Pi })(1-\omega ) \Pi _{b,t} - BD_t \end{aligned}$$

where $\Pi _{b,t}$ is the bank’s profit and $BD_t$ is *bad debt*, i.e., the book value of non-performing loans.

### A.5 Entry-exit mechanism

If the liabilities of a firm exceed its assets (so that its equity turns negative), it is assumed to go bankrupt and exit.^16^

A newly created firm will enter sector $j=B,L,K$ and replace a bankrupt firm in the same sector with probability $\pi ^e_{j,t}=\left[ 1+\exp (\gamma _e \kappa ^j_t)\right] ^{-1}$, which increases with the average profit rate prevailing in the sector $\kappa ^j_t$. The firm owner of the previously bankrupt firm being replaced will provide the initial equity injection to the entrant firm. In a sense, a firm which goes bankrupt is ‘dormant’, remaining inactive for a variable number of periods until a new firm succeeds in entering and replacing it. At any given time, therefore, the number of *active* firms may be smaller than $N_F$. $N_F$ itself is fixed and the number of active firms cannot exceed it.

Regarding the bank, we assume that if its equity becomes negative due to losses on bad debt, a bail-in procedure will immediately be applied: all firm-owners (who collectively own the bank) make a transfer to the bank to make its equity positive again.

### A.6 The public sector

The public sector taxes wages and profits, provides unemployment subsidies, sick-pay and pensions (to workers), makes interest payments on government bonds (to the bank) and carries out government expenditure on healthcare. The latter is a constant fraction *g* of full employment output, taking the initial population of active workers $N_A$ as a basis for calculation. In symbols:

A.20 $$\begin{aligned} G=g \alpha _N N_{W} \end{aligned}$$

We assume this expenditure is used to purchase both C-goods and K-goods and translates one for one into a supply of healthcare services to the population via the public healthcare system. *G* is in the first instance allocated to firms according to their relative revenue in the previous period.^17^

A public sector deficit occurs when taxes turn out to be lower than the sum of transfer payments, interest payments and government expenditure on healthcare. In this case, the government will issue new bonds. For simplicity, we assume that all government bonds are purchased by the bank at the fixed risk-free rate *r*.

### A.7 Demand and supply of healthcare

We assume that economic decisions are taken every month while the health component of the model runs at a weekly frequency. We will indicate the current week with the subscript $\tau$.

We assume that, in any given week, a healthy agent may catch a non-infectious disease with a certain probability $\pi ^i$. The presence of this disease in turn generates a demand for healthcare services. The non-infectious disease is also not lethal: after a fixed number $D^n_d$ of weeks the sick recover. Recovery does not imply immunity: recovered agents may randomly become susceptible again in the future. This assumption implies that the non-infectious disease is endemic.

For simplicity we assume that only workers (both active and inactive) may get ill with the non-infectious or the epidemic disease (described below). Since, as outlined below, age is an important factor affecting the course of the epidemic disease, we divide the population of workers into three age-segments. For simplicity we assume that agents do not age, i.e. they remain in the age-segment to which they are assigned. We denote with $\phi _y$, $\phi _m$ and $\phi _o$ the fractions of the population consisting of young, middle-aged and old workers and calibrate them such that their values roughly capture the current composition by age of the population of Lombardy. The variable $age_h$ assumes value 1 if the agent is young – i.e., if they belong to the fraction $\phi _y$ of the population – 2 if middle-aged and 3 if old. The h-th sick agent, $h\in (1,N_W)$, generates a demand of healthcare in week $\tau$ – denoted with $H^d_{h,\tau }$ which is increasing with age and affected by an idiosyncratic shock:

A.21 $$\begin{aligned} H_{h,\tau }^d=h_1 age_{h}+h_2 u_{h,\tau } \end{aligned}$$

where $$u_h \sim \mathcal {U}(0,1)$$.

The total supply of healthcare services in every period $\tau$ is given by *G*. In the first instance, this is allocated to agents who were already ill and receiving healthcare in the previous period and who still require it. The residual is then allocated to patients who have fallen ill in the current period: A randomised queue of all agents requiring and not already receiving healthcare is formed and agents are admitted into the healthcare system until the residual supply is exhausted. Hence the demand for healthcare may be rationed. If an agent’s demand exceeds the remaining supply of healthcare, that agent is rationed and receives only a fraction of the desired supply. All subsequent agents in the queue are rationed completely. All rationed agents will queue again in the next period if they still require care.

Sick agents who were previously in the labor force become inactive and receive sick-pay. Old people are inactive by assumption and receive pensions. Retired agents who become sick will continue to receive the pension instead of sick-pay.

## Appendix B: The epidemiological sub-model

In this appendix we describe the dynamics of an epidemic caused by an *infectious* disease. The epidemic differs from the non-infectious disease because of the transmission from one subject to the others through *contagion*. Despite being based on contagion through a network structure of contacts between agents, the epidemiological sub-model is similar to classic compartmental models in that agents can be classified into various states. The notation is as follows:

$\mathcal {I}_{\tau }$ denotes the *cumulative* number of (both detected and undetected) infections from the beginning of the epidemic up to period $\tau$. $\dot{ \mathcal {I}}_{\tau }$ denotes the number of *new* infections in $\tau$. $\mathcal {I}_{c,\tau }$ denotes the stock of *currently* infected agents in $\tau$. $\Delta \mathcal {I}_{c,\tau }$ denotes the *change* in the stock of currently infected in $\tau$. Note that in general, $\Delta \mathcal {I}_{c,\tau } \ne \dot{ \mathcal {I}}_{\tau }$, since the former includes newly recovered agents (and hence may be negative) whilst the latter only includes new infections and hence must be $\ge 0$. Similarly, let $\mathcal {D}_{\tau }$ denote the cumulative number of *detected* infections, with $\dot{ \mathcal {D}}_{\tau }$, $\mathcal {D}_{c,\tau }$ and $\Delta \mathcal {D}_{c,\tau }$ having the equivalent interpretations of the variables defined above. $\mathcal {M}_{\tau }$ is the cumulative number of deaths, $\mathcal {H}_{\tau }$ the cumulative number of agents requiring healthcare due to the epidemic disease, and $\mathcal {R}_{\tau }$ the cumulative number of recoveries from the epidemic disease. For all three, we also define the respective derivative variables as above.^18^ Finally, $\mathcal {S}_{c, \tau }$ denotes the stock of agents who are currently susceptible to the disease and $\Delta \mathcal {S}_{c,\tau }$ the change in this stock. The values of all epidemiological model parameters introduced below can be found in Appendix E.

The epidemic begins in an exogenously determined week labelled $\tau _E$, in which a small number of workers are exogenously infected with the epidemic disease. These people are the *initial infected* (and infectious) and will be denoted with $\mathcal {I}_{c,\tau _E}$. The (healthy and) susceptible agents after the appearance of the infected are $\mathcal {S}_{c,\tau _E}=N_{W}-\mathcal {I}_{c,\tau _E}$ since at the beginning of the epidemic, all $N_W$ workers in the model are alive. These susceptibles may then be infected by the initial infected in $\tau _E$ and subsequent periods as described below.

Some infected agents develop mild symptoms or do not develop symptoms at all (*non-symptomatic* for short). In this case the infection can be detected only if the agent is subjected to a test. In each period, every undetected infected agent may be detected with a probability $\pi ^r_\tau$ which, as explained in the main text, becomes endogenous as the epidemic progresses. Agents who test positive are quarantined and therefore cannot spread the disease. People who develop serious symptoms are detected and quarantined with certainty. Therefore, the disease is only spread by non-detected infected agents. $\mathcal {D}_{\tau }$, the cumulative number of detected infections, includes all infections leading to serious symptoms as well as all infections detected through tests on non-symptomatic agents. The probability for an agent to develop serious symptoms is increasing with age. All agents developing serious symptoms require healthcare and hence become part of $\mathcal {H}_{\tau }$. Their individual demand for healthcare is given by Eq. A.21. All agents who are currently infected and detected, $\mathcal {D}_{c,\tau }$ will be inactive (and receive sick pay if they are not retired) and will not have contacts with other agents for the entire duration of the disease. For simplicity, we assume that the infected remain contagious for the entire duration of the illness. The undetected infected therefore can spread the disease for the entire duration of their illness.

Non-symptomatic agents recover with certainty after a certain number of periods of being ill, with the duration of the infectious disease – denoted with $d^i$ – being drawn from a uniform distribution for any infected individual. Agents developing serious symptoms, on the other hand, may die with some probability during each period of the illness before recovering. In period $\tau$, the *h*-th infected agent with serious symptoms will face a probability of death which is increasing with age and with excess demand for health care:

B.1 $$\begin{aligned} \pi ^m_{h,\tau }=\widehat{\pi }^m_{\tau } age_h^3+h_{3,\tau }(H_{h,\tau }^d-H_{h\tau }^s) \end{aligned}$$

where $\widehat{\pi }^m_{\tau }>0$ and $h_{3,\tau }>0$, $H_{h,\tau }^d$ is the agent’s demand for health care and $H_{h,\tau }^s$ is the amount of healthcare they actually receive, which depends on the free capacity of the healthcare system. We assume that both $\widehat{\pi }^m_{\tau }$ and $h_{3,\tau }$ decrease over time even in the absence of a vaccine until they reach a lower bound. A rationale for this assumption is that even without a vaccine, healthcare systems may over time become better at treating a novel disease (in the case of Covid-19 this may involve the use of existing or new medicines other than vaccines, increasing experience as to when patients should be intubated, etc.). The laws of motion are:

B.2 $$\begin{aligned} \begin{aligned} \widehat{\pi }^m_{\tau }&=\max (\underline{\pi ^m},\widehat{\pi }^m_{\tau -1}(1-z))\\ h_{3,\tau }&=\max (\underline{h_{3}},h_{3,\tau -1}(1-z)) \end{aligned} \end{aligned}$$

Instead of postulating the law of motion of the number of infected people as in SIR models, we adopt a granular approach to contagion focusing on networks in order to depict the transmission of the epidemic among agents.

Contagion takes place via three networks: the workplace (employment network), the marketplace (shopping network) and social relations (social network). Employed workers are nodes in the *employment network*. Each employed worker is linked to all co-workers in the firm for which they work, meaning that they encounter them every week. If a firm goes into smart working, only a share of possible workplace encounters take place. If a firm is shut down by a lockdown, no workplace encounters occur at that firm.

In addition, all worker households are nodes in the *shopping network*. A certain number of households shop at a given C-firm. If one of these buyers is infectious, they can spread the disease to other households shopping at the same firm. We list all possible connections between the customers of a given firm and assume that a fixed share ($\frac{1}{3}$) of those encounters actually take place (reflecting the assumption that not all customers visit the firm at the same time). This share is reduced if there is a lockdown in place or people engage in social distancing.

Finally, we build a *social network* to depict encounters during leisure time. Each worker household has a set of social connections consisting of family and friends. The total number of social connections is a (very small) fraction of the maximum number of possible undirected connections between worker households, $\frac{N_{W}(N_{W}-1)}{2}$.

While both the employment and shopping networks change over time as households change employment and the firms at which they shop, the social network is static.

We assume that each infected and undetected agent meets all the agents they are connected to (at work, while shopping and during leisure time) in every week. Let $N^{\mathcal {C}}_\tau$ denote the number of connections in week $\tau$ which involve exactly one undetected infected and one susceptible agent. We assume that only a fraction (the transmission rate) of these connections may lead to a new infection. In other words, there is a *maximum number* of potential *new* infections in week $\tau$ given by

B.3 $$\begin{aligned} \dot{\mathcal {I}}_{\tau }^{\max }=\rho _{c,\tau } N^{\mathcal {C}}_\tau \end{aligned}$$

where $\rho _{c,\tau } \in (0,1)$ is the transmission rate which incorporates a seasonal effect, being lower from May to September (late spring and summer in the Northern hemisphere) and higher from October to April (autumn, winter and early spring in the Northern hemisphere). We then take a sample of size $\dot{\mathcal {I}}_\tau ^{\max }$ from the set of connections involving exactly one undetected infected and one susceptible agent. The sample is weighted such that the likelihood of being drawn is highest for social connections, second-highest for employment connections and lowest for shopping connections. In the absence of social distancing, each of these connections leads to an infection with certainty. Under social distancing, a connection leads to an infection with probability $1-\beta$ if one agent involved in the connection is socially distancing and with probability $1-2\beta$ if both agents are socially distancing.

As indicated above, the infection with the epidemic disease ends either with recovery or death. If an infected agent recovers, the stock of currently recovered agents, $\mathcal {R}_{c,\tau }$ increases by one. If the agent was previously economically active and became inactive due to their infection being detected, they will re-enter the labor force as an unemployed agent and begin to look for a job. If an infected agent dies, the stock of current dead, $\mathcal {M}_{c,\tau }$ increases by one. We assume that there are no bequests, such that the assets of dead agents are simply written off. Recovered agents eventually become susceptible to the disease again and dead agents can be replaced by newly born ones. Once an agent recovers, they become fully immune to the epidemic disease for a number of weeks which is given by a draw from a normal distribution $\mathcal {N}(52,2)$.^19^ After this number of weeks has passed, the agent becomes susceptible. An agent may hence be infected with the epidemic disease more than once over the course of a simulation run. This factor makes it more likely for the disease to become endemic. At the beginning of every month *t*, every dead agent may be replaced with a new (and susceptible) agent belonging to age group 1 (i.e. a young agent) with a low probability $\pi ^n$ ($=0.0125$ in the simulations shown here). If the dead agent is replaced, the stock of currently dead agents decreases by one. This replacement mechanism ensures that when the disease becomes endemic, it does not cause the entire population to eventually die out. Since the probability of death never goes to zero, the absence of a replacement mechanism for dead agents would mean that unless the disease dies out first, the model population would go to zero in the very long run (i.e. beyond the time horizons simulated in this paper).

### B.1 Social distancing

Endogenous social distancing (SD) is modelled as a discrete choice by individual agents (see also Baskozos et al. 2020). We define a *distancing index* $d_{\tau }$ which is governed by the law of motion:

B.4 $$\begin{aligned} d_{\tau }=\iota d_{\tau -1}+(1-\iota )\textbf{N} \times {\textbf {D}}_\tau , \end{aligned}$$

where $\textbf{N}$ is a row vector containing three parameters describing the intensity of choice and ${\textbf {D}}_\tau$ is a column vector containing three indicators influencing agents’ decision to distance. The first is a measure of the severity of the epidemic, given by $\mathcal {D}_{c,\tau }-\overline{\mathcal {D}}_{c,SD}$ where $\mathcal {D}_{c,\tau }$ is the number of *currently* infected *and detected* individuals and $\overline{\mathcal {D}}_{c,SD}$ is a fixed threshold value. The second captures social influence and is given by $\phi _d-\phi _{nd}$, that is the difference between the share of agents which are already socially distancing ($\phi _d$) and those who are not ($\phi _{nd}$). The third is a perceived cost of social distancing, denoted by $c_{SD}$ which is multiplied by $-1$. The probability for an agent to distance is then given by

B.5 $$\begin{aligned} \pi _{\tau }^d=\frac{1}{1+\exp (-d_{\tau })} \end{aligned}$$

so that the probability of distancing is increasing in the index *d* and asymptotically tending to 1 for $d_{\tau } \rightarrow \infty$. In words: an agent is more likely to distance (i) the higher the number of currently detected cases, (ii) the higher the fraction of agents who are already distancing, and (iii) the lower the perceived cost of social distancing.

In each period we draw a random number $n_h$ from a uniform distribution $\mathcal {U}(0,1)$ for each agent and if $\pi ^d_{\tau }>n_h$, the agent will engage in social distancing. By assumption, social distancing has three important effects on both the economic and the epidemiological dimensions of the model.

First of all, social distancing affects the probability of infection conditional on an encounter between a susceptible and an infected individual. In the absence of social distancing, a fraction (transmission rate) of meetings between susceptible and infected agents leads to a new infection with probability 1. By assumption, with social distancing a meeting between an infectious agent *i* and a susceptible agent *j* generates an infection with probability

B.6 $$\begin{aligned} \pi ^d_{i,j}=1-\beta \textbf{1}_{\pi ^d_{\tau }>n_i}-\beta \textbf{1}_{\pi ^d_{\tau }>n_j} \end{aligned}$$

where $\textbf{1}_{\pi ^d_{\tau }>n_z}$ is an indicator function which takes value 1 if agent *z* is socially distancing and 0 otherwise, $z=i,j$. For each of these meetings, therefore, the probability of infection goes down to $1-\beta$ if one of the agents involved is distancing, and $1-2\beta$ if both are distancing.

Second, SD reduces the overall number of connections. In the presence of social distancing, for all $t>t_E+2$, the number of social connections and shop connections is reduced by $(1-\phi _d)$.^20^

Thirdly, social distancing affects agents’ demand for consumption goods. We assume that the first time any agent socially distances, their demand for L-goods (resp. B-goods) receives a negative (positive) shock. The shocks are calibrated such that in percentage terms, the demand for luxury goods declines at a faster pace than the pace at which the demand for basic goods increases. In this way we capture two well known stylized facts of the epidemic: the fall in (aggregate) consumption and the change in the composition of the consumption basket in favor of basic/essential goods. Finally, we assume that the process of contraction of the consumption basket and change of its composition is gradually fading away.

In symbols, the shocks are given by

B.7 $$\begin{aligned} \begin{aligned} s_B&=1+\sigma ^B_{\tau }\mathcal {D}_{c,\tau }\\ s_L&=1-\sigma ^L_{\tau }\mathcal {D}_{c,\tau }, \end{aligned} \end{aligned}$$

where

B.8 $$\begin{aligned} \begin{aligned} \sigma ^B_{\tau }&=\max (\underline{\sigma }^B,\overline{\sigma }^B*\exp (-z*\widehat{\tau }))\\ \sigma ^L_{\tau }&=\max (\underline{\sigma }^L,\overline{\sigma }^L*\exp (-z*\widehat{\tau })), \end{aligned} \end{aligned}$$

The dynamics described by Eq. B.8 captures the decline over time of the size of the consumption shock as households become accustomed to the new epidemic environment. Of course $\overline{\sigma }^B<\overline{\sigma }^L$ and $\underline{\sigma }^B<\underline{\sigma }^L$. The time index $\widehat{\tau }$ is equal to 0 (as long as $t\le t_E+2$), and then increases by 1 in each week.

### B.2 Lockdown

A one-off government mandated lockdown comes into force if the number of newly detected infections during a week (denote this as $\tau ^s_L$) reaches the (exogenous) threshold $\dot{\mathcal {D}}_{lock}$. If the situation does not improve, the lockdown ends after a maximum duration of $d^{\max }_{lock}$ weeks. If the situation improves prior to $\tau ^s_L+d^{\ max}_{lock}$ – i.e., if the average of newly detected cases over the previous 2 weeks falls below a floor $\dot{\mathcal {D}}_{end}$ – the lockdown is lifted. We denote the period in which the lockdown ends with $\tau ^e_L$. We assume that the lockdown is enforced only once. In other words, if a lockdown has been imposed and subsequently lifted, there are no subsequent lockdowns even if detected infections rise beyond the threshold again in future periods. The lockdown triggers the following events in the model:

- At the beginning of the month following the institution of the lockdown, a fraction of firms producing luxury/inessential goods (L-firms) are shut down completely (and their production is halted). They become unable to sell any goods they may have already produced during that period. All firms which are closed in lockdown lose all of the customers who are using them as their “go-to” firm. Firms producing basic goods and capital goods (B-firms and K-firms respectively) continue producing.
- All firms immediately move into “smart working” meaning that a subset of workers of each firm work from home. This eliminates part of the connections at the workplace: the number of connections at every firm in smart working is reduced by a fixed factor (uniform across firms). The exact subset of connections which does take place is sampled anew in every week. The L-firms which are closed completely do not give rise to any workplace connections during the lockdown.
- The lockdown limits social gatherings, eliminating a part of the connections in the social network of agents. Hence the number of social connections is a fraction of the corresponding number in normal times. Connections occurring in the shopping network are also reduced by the same factor. This may be interpreted as agents making fewer shopping trips than they otherwise would and, for instance, increasingly relying on deliveries of goods. In addition, we assume that the lockdown lowers the perceived cost of social distancing, making it more likely, ceteris paribus, that any individual agent will engage in social distancing.
- Once the lockdown has ended, previously closed firms are re-opened but remain in smart working mode. Expected demand is re-initialised for all L-firms to account for the entry of the reopening firms.^21^ After the lockdown, each firm moves out of smart working after a stochastic number of periods. Encounters between agents slowly adjust back to their previous level, as does the perceived cost of social distancing.
- We assume that the lockdown is associated with an increased effort to detect infections. Once $\tau >\tau ^e_L$, the previously exogenous probability of detecting an asymptomatic infected agent becomes endogenous and time-varying. In particular, the detection probability in week $\tau$ is then given by $\pi _{\tau }^r=\min (\overline{\pi ^r}, \underline{\pi ^r}+\gamma _d \dot{\mathcal {D}}_{\tau -1})$, where $\dot{\mathcal {D}}_{\tau -1}$ is the number of newly detected cases in the previous week and $\overline{\pi ^r}$ is an upper bound.

The effects of the lockdown on the network of connections between agents is illustrated using Figs. 10 and 11 which show an example of the network (encompassing all three types of connections, i.e., workplace, shops and social) during one period in the absence of an epidemic and one in lockdown. The reduced number of connections is immediately obvious, and is also demonstrated by an examination of two simple measures of connectivity. The network depicted in Fig. 10 has a density of 0.0021 and the largest eigenvalue of the corresponding adjacency matrix is equal to 66.11. In Fig. 11, by contrast, the density has declined to 0.00027 and the largest eigenvalue is equal to 25.05.

### B.3 Vaccine effectiveness and strategies

As in reality, immunity to the epidemic disease in our model can take two forms: natural immunity acquired by means of infection and recovery; vaccine-induced immunity generated by vaccination. Vaccination may have three distinct effects: it may reduce (i) the susceptibility of vaccinated individuals to infection; (ii) the exposure of vaccinated individuals to serious disease (and hence mortality); (iii) the transmissibility of the infection by a vaccinated individual who has become infected. Vaccine Effectiveness^22^ may hence be measured with respect to:

1. susceptibility to infection ($VE_1$)
2. vulnerability to serious disease and death ($VE_2$)
3. transmissibility of infection ($VE_3$)

With regard to vaccines against Covid-19, there is ample evidence that vaccine-induced immunity of type 1 is not complete: the estimates vary from around 60 to around 90% depending on the vaccine (lower for viral vector vaccines, higher for mRNA vaccines). Hence, a vaccinated person may still be infected, but with a lower probability. To capture this feature, we assume that when an infectious agent *i* meets a susceptible agent who has received the vaccine *v*, the probability $\pi _{i,v}^{tr}$ that *i* will transmit the disease to *v* is reduced by $VE_1$ and set $VE_1=0.8$. The probability of transmission in case of vaccination, therefore, is described by a modified Eq. B.6, namely:

B.9 $$\begin{aligned} \pi ^{tr}_{i,v}=(1-VE_{1}\textbf{1}_{\tau ,v})(1-\beta \textbf{1}_{\pi ^d_{\tau }>n_i}-\beta \textbf{1}_{\pi ^d_{\tau }>n_v}) \end{aligned}$$

where $\textbf{1}_{\tau ,v}$ is an indicator function which takes value 1 if *v* is vaccinated and 0 otherwise.

Clinical evidence suggests that the existing vaccines’ ability to prevent serious disease ($VE_{2}$) is sizable and somewhat higher than $VE_{1}$; estimates range from 90 to 95%.

In the model, the subset of agents who develop serious symptoms if infected is defined at the beginning of the simulation.^23^ Agents in this subset will develop serious symptoms with a probability equal to 1 in the absence of vaccinations. We assume that if an individual belonging to this subset is vaccinated, they will develop serious symptoms only with probability $\pi ^h_v=1-VE_{2}$ and set $VE_2=0.95$

On the basis of the evidence collected so far, it is unclear whether Covid-19 vaccines are also effective in reducing the probability that an infected but vaccinated individual infects other people. Estimates of $VE_{3}$ vary considerably across studies. We assume the worst case scenario of vaccinated infected individuals being as infectious as unvaccinated ones: $VE_3=0$.^24^

We assume that a single dose of the vaccine provides the levels of immunity given above, for a duration which (in weeks) is given by a random draw from a normal distribution with mean equal to 52.^25^ Thereafter the protection disappears ($VE_1=VE_2=VE_3=0$) and the individual goes back to the status of susceptible. For the moment we rule out the presence of people who refuse to get vaccinated: all individuals eligible for vaccination accept the vaccine when it is offered to them.

There are two key features which characterize a vaccination strategy:

- the *coverage rate*, i.e., the fraction of the population which can be vaccinated in one unit of time;
- the *priority rule*, i.e., the procedure defining which target groups (if any) should be prioritized for vaccination, e.g. old or young.

Both dimensions can affect the efficacy of the vaccination campaign, the evolution of the epidemic and the resulting effects on the macroeconomy.

We define the coverage rate as the share of the population which can be vaccinated *in every week*. This may be influenced by vaccine availability as well as the technical and organizational capacity of the vaccination authorities, both of which should improve over time. In the simulations, in order to replicate the actual time-line in Italy, we assume that the vaccine is introduced 11 months after the outbreak of the epidemic (corresponding to December 2020) – i.e., well after the end of the first lockdown. We also assume that the coverage begins at a low level (0.01) and then increases by 0.001 in every period until reaching an upper bound (0.05). The pool of agents eligible for the vaccine consists of susceptible, recovered and undetected infected agents. The order in which agents are selected from the pool in a given period is defined by the priority rule. The latter may be designed in a way to place more weight on certain conditions than others, say health-demographic versus economic factors, depending on the particular strategy adopted by the policy maker.

We compare three alternative vaccination strategies based on different priority rules: Randomized Vaccination (RV); Priority by Age (PA); Priority to Workers (PW). In the RV strategy, agents to be vaccinated are randomly sampled with equal probability from the pool of eligible individuals in each period. In the PA strategy, the probability of being drawn from the pool increases (exponentially) with age, thus giving priority to the old. Finally, in the PW strategy, the probability of being drawn is substantially higher for active workers – that is young and middle-aged agents – than for the inactive old.^26^ As the vaccination campaign progresses, however, the strategies become equivalent once the number of available doses per week eventually exceeds the number of remaining unvaccinated agents.

### B.4 Modelling of variants

Variants may enhance the infectiousness of the virus and/or its capability of causing severe disease and death. One feature which variants of concern of SARS-CoV-2 so far have had in common is enhanced transmissibility with respect to the original virus, while evidence on changes in mortality to date is mixed (also depending on the particular variant). Importantly, variants may also reduce vaccine effectiveness, both in preventing infection and in avoiding serious disease (Zimmer 2021).

As outlined in the main text, we model two types of variant. We assume that both variants are more contagious than the original virus but do not affect mortality: their transmission rate $\rho _c$ is 50% higher and the effectiveness of social distancing $\beta$ is 75% lower than for the original virus; the fatality rate, $\pi ^m$, instead, is not affected by the emergence of the variant. The variants differ only because of their type of resistance to the vaccine against the original virus. While Variant 1 is able to reduce only the vaccine’s ability to prevent infection – i.e., $VE_1$ – Variant 2 reduces both $VE_1$ and $VE_2$, i.e., also the vaccine’s effectiveness in preventing serious disease which may eventually lead to death.

Since both variants which we model are identical in terms of infectiousness, their initial diffusion dynamics are very similar. We hence focus on the scenario of Variant 1 in the absence of an improved vaccine in describing the spread of a variant. Starting from the PA scenario (i.e. an epidemic with subsequent introduction of a vaccine against the original virus, with priority given to old agents), the variant is introduced in the model by assuming that a small number of “super-spreaders” are exogenously infected with the variant a few weeks before the start of the vaccination campaign. These “super-spreaders” are chosen as the 5 currently infected individuals with the highest number of connections to susceptible agents. The infection status of these 5 agents is exogenously changed from the original virus to the variant, which they subsequently spread among their contacts. At first the variant can coexist with the original virus, implying that susceptible agents may be infected with either one of the two. Given the higher transmission rate, however, the variant becomes dominant fairly quickly and endogenously replaces the original virus.

The diffusion of Variant 1 in this scenario is illustrated in Fig. 12. In the figure we consider a time window consisting of 200 weeks starting a few weeks before the outbreak of the epidemic. The solid line shows the total flow of new infections per week due to both the original virus and the variant once it is introduced. The dotted line represents the flow of new infections due exclusively to the variant, which is introduced right around the peak of the second wave. Over time the variant supplants the original virus. The dynamics of the epidemic are subsequently characterized by a sequence of waves of infections, similar in amplitude to those produced by the scenario without any vaccine, despite a vaccine against the original virus being present in this scenario. The variant hence plays the role of a *contagion accelerator* leading to an increase of the amplitude of the waves of infection.

## Appendix C: Calibration of the macroeconomic sub-model

In order to calibrate the parameters for the macroeconomic sub-model, we make use of data for real GDP, consumption, gross fixed capital formation and employment for the Lombardy region of Italy from 1995 to 2017, available from the website of the Istituto Nazionale di Statistica (Istat).^27^

At the regional level, data for GDP and its components are available only at annual frequency. We use moments and statistics calculated from these data in order to calibrate and validate the model. We apply the HP filter ($\lambda =100$) to the empirical time series and then calculate the standard deviations (relative to the trend component) and autocorrelations of the filtered series. Table 2 shows the empirical statistics we obtain as well as 95% confidence intervals (in parentheses) which we generate using bootstrapping.

After identifying a region of the parameter space in which the model gives rise to reasonable macroeconomic dynamics, several parameters are fine-tuned in order to replicate the moments and statistics shown in Table 2 with the simulated time series. The values of many parameters turn out to be quite similar to those shown in Delli Gatti and Reissl (2022) while others differ somewhat. The full set of parameter values is shown in Table 4 in Appendix E.

The model is simulated 100 times with different seeds for a duration of 756 periods, equivalent to 63 years. Simulated monthly time series are transformed into annual ones and then filtered in order to construct the simulated equivalents of the empirical moments and statistics. The simulated moments are shown in Table 3 as means across Monte Carlo runs along with the associated confidence intervals.

As in Delli Gatti and Reissl (2022), the model is able to closely reproduce the empirical standard deviations of GDP and consumption. Simulated investment however is significantly more volatile than its empirical counterpart. Since simulated GDP consists solely of private consumption and investment (along with constant public expenditure for healthcare) in our model and there is no role for net exports, the empirical volatility of GDP and consumption can only be reproduced if simulated investment is more volatile than empirical investment.

**Table 2:** Empirical moments & statistics for Lombardy (1995-2017)

Statistic	GDP	Consumption	Investment	Employment rate
Std	0.015722	0.01488	0.05100	0.00711
deviation	(0.01566; 0.01578)	(0.01485; 0.01491)	(0.05087; 0.05113)	(0.00708; 0.00713)
1st order	0.15342	0.40094	0.39838	0.42836
autocorr	(0.14964; 0.15720)	(0.39885; 0.40303)	(0.39562; 0.40115)	(0.42574; 0.43098)
2nd order	$-$0.12215	$-$0.24278	0.03476	$-$0.05605
autocorr	($-$0.12637; $-$0.11793)	($-$0.24566; $-$0.23990)	(0.03174; 0.03778)	($-$0.05958; $-$0.05253)

Similarly, the simulated employment rate is much more volatile than in the empirical data. This is due to the simplified nature of the labor market in our model which leads to employment being tied to current production much more closely than it is in reality.

**Table 3:** Simulated moments & statistics

Statistic	GDP	Consumption	Investment	Employment rate
Std	0.01564	0.01524	0.09254	0.01536
deviation	(0.01466; 0.01662)	(0.01432; 0.01616)	(0.08738; 0.09769)	(0.01441; 0.01632)
1st order	0.59272	0.58364	0.47737	0.59020
autocorr	(0.56285; 0.62259)	(0.55346; 0.61381)	(0.44081; 0.51393)	(0.56015; 0.62025)
2nd order	0.07775	0.05158	0.04948	0.07511
autocorr	(0.03137; 0.12412)	(0.00415; 0.09901)	(0.00680; 0.09217)	(0.02866; 0.12157)

The model does reasonably well at reproducing most of the first order autocorrelations but performs less well on second and higher-order autocorrelations especially regarding GDP and consumption. Similarly to Assenza et al. (2015) and Delli Gatti and Reissl (2022), we plot the autocorrelations of output, consumption, investment and the employment rate up to lag 6 in Figs. 13, while 14 shows the cross-correlations of output, consumption, investment and the employment rate with output.

## Appendix D: Additional simulation experiments

In this appendix, we present some additional computational experiments, the first one of which may be of interest from a policy perspective, with the latter two serving as sensitivity analyses on two of the most important parameters of the epidemiological sub-model.

### D.1 Effects of increased healthcare expenditure

Taking the EP scenario shown in Section 3, i.e. a setting with an epidemic and an endogenous lockdown but no vaccination and no virus mutation, as the baseline, we investigate the effects of an increase in government healthcare expenditure at the beginning of the epidemic. In all scenarios shown above, government healthcare expenditure in real terms is fixed to 4% of full employment GDP. In the experiment, we permanently increase this expenditure to 5% of full employment GDP one month after the epidemic disease is introduced in the model and compare both epidemiological and economic outcomes to those observed in the baseline.

The increase in healthcare expenditure translates directly into an equivalent increase in the per-period supply of healthcare services and hence the capacity of the healthcare system to treat patients. The experiment hence abstracts from any time-lags or possible difficulties in quickly increasing healthcare capacity, but should nevertheless give an idea of the possible benefits.

Figure 15 shows the epidemiological effects of increased healthcare expenditure, comparing the series for infections and deaths for the baseline scenario from Fig. 1 to those resulting from the experiment. While the increase in healthcare expenditure does not have any significant effect on the number of infections, it dramatically decreases the number of deaths caused by the epidemic through reducing the incidence of shortages in the provision of healthcare services.

Figure 16 gives a brief overview of the macroeconomic effects of the increase in healthcare expenditure, showing the dynamics of real GDP and government debt as a ratio of nominal GDP relative to the scenario without an epidemic, as in Fig. 4. While the increase in healthcare expenditure initially has a positive effect on GDP compared to the baseline scenario due to increased government demand, this increase is eventually balanced out as the increase in healthcare capacity chiefly prevents the deaths of old and hence economically inactive agents (who are therefore not replaced by economically active ones). The increased government expenditure for healthcare combined with the pension expenditures for surviving old agents also give rise to a government debt to GDP ratio which is higher than in the baseline scenario.

### D.2 Post-infection immunity

Recall that if an agent is infected with the epidemic disease and subsequently recovers, they subsequently become immune to the epidemic disease for a number of weeks given by $\mathcal {N}(52,2)$. As a mean value of 52 may be regarded as somewhat high, we conduct a sensitivity analysis by re-running the EP scenario shown in Section 3, i.e. a setting with an epidemic and an endogenous lockdown but no vaccination and no virus mutation, with the values 39, 26 and 13 for this parameter.

Figure 17 shows that while the number of deaths does not appear to be particularly sensitive to the choice of this parameter, it does have considerable effects on the number of infections, especially following the second wave of the epidemic, with subsequent waves increasing both in terms of length and magnitude, particularly for low values of the parameter.

This change in epidemiological outcomes following the second wave is also strongly reflected in the macroeconomic effects of the epidemic, as shown in Fig. 18. Subsequent large waves of infections give rise to concurrent increases in endogenous social distancing and associated declines in economic activity, producing dynamics resembling a limit cycle as well as a generally lower level of GDP. These effects on economic activity are also reflected in a higher ratio of government debt to GDP.

### D.3 Vaccine-induced immunity

Recall that if an agent is vaccinated, their probability of becoming infected with the epidemic disease and, if infected nevertheless, to develop serious symptoms, is decreased for a number of weeks. As in the case of post-infection immunity, this duration is drawn from $\mathcal {N}(52,2)$ and, as in the case of the former, a mean duration of 52 may be regarded as somewhat high. We hence conduct a similar sensitivity analysis to the one shown in the previous sub-section. Taking the PA scenario, i.e. a setting with vaccination and prioritisation by age but no virus variants, as our point of departure, we re-run the model with the values 39, 26 and 13 for the mean duration of vaccine-induced immunity and compare the outcomes to those for a value of 52.

Figure 19 summarises the epidemiological outcomes, starting at the beginning of the vaccination campaign one year after the epidemic disease is introduced in the model. It can be seen that significant epidemiological effects arise only in the case of the lowest mean duration of vaccine-induced immunity. In this latter case, however, infections and especially deaths increase substantially compared to the other three scenarios. As the mean duration of vaccine effectiveness decreases, the number of agents which require re-vaccination in any given period increases; if the duration becomes sufficiently short, this number will exceed the capacity of the system to administer new doses, leading to the presence of unvaccinated agents who are more vulnerable to the disease.

Figure 20, which plots the dynamics of GDP and the government debt ratio relative to the epidemic scenario without vaccination, however, shows that even when the duration of vaccine effectiveness takes its lowest value, vaccination still has a positive - though slightly smaller - effect on economic activity. Similarly, the availability of a vaccine always gives rise to a lower government debt to GDP ratio, though this decrease is somewhat smaller when the duration of vaccine effectiveness takes its lowest value.

## Appendix E: Parameter values

Tables 4 and 5 below provide the lists of model parameters pertaining to the macroeconomic sub-model and the epidemiological sub-model respectively.

**Table 4:** Macroeconomic sub-model parameters

Symbol	Description	Value
$N_{W}$	Number of workers	30000
$N_{F}^{b}$	Number of B-firms	360
$N_{F}^{l}$	Number of L-firms	240
$N^{k}_{F}$	Number of K-firms	200
$z_{e}$	Number of Firms visited by unemployed	5
${\xi }_{Y}$	Memory parameter for human wealth	0.55
$c_{W}$	Propensity to consume out of financial wealth	0.00835
${\rho }_{q}$	Quantity adjustment parameter	0.2
$\overline{\rho _p}$	Price adjustment upper bound	0.08
$\mu$	Bank’s gross mark-up	1.007
$\delta$	Capital depreciation rate	0.01
$\pi ^k$	Probability to invest	0.4
$\phi _b$	Bank’s leverage parameter	0.0025
$\zeta$	Debt repayment rate	0.05
$\xi _K$	Memory parameter for capacity utilisation	0.2
$\alpha _N$	labor productivity	2/3
$\alpha _K$	Capital productivity	1/6
$\omega$	Dividend payout ratio	0.25
$\overline{x}$	Target capacity utilisation	0.85
$\delta ^k$	Inventory depreciation	0.08
$b_{0c}$	Bank’s risk evaluation parameter (C-firms)	-10
$b_{1c}$	Bank’s risk evaluation parameter (C-firms)	10
$b_{0k}$	Bank’s risk evaluation parameter (K-firms)	-15
$b_{1k}$	Bank’s risk evaluation parameter (K-firms)	15
*r*	Risk-free interest rate	$\frac{0.01}{3}$
$r_d$	Interest rate on deposits	$\frac{r}{2}$
$s_u$	Replacement rate (unemployment subsidy)	0.75
$s_p$	Replacement rate (pension)	0.9
$s_s$	Replacement rate (sick-pay)	0.75
$t_w$	Tax rate on wage income	0.275
$t_{\Pi }$	Tax rate on profits	0.3
$u^{up}$	Upward wage adjustment parameter	$\frac{0.1}{3}$
$u^{down}$	Downward wage adjustment parameter	$\frac{0.01}{3}$
$u^T$	Unemployment threshold	0.1
*g*	Ratio of government healthcare expenditure to full employment GDP	0.04
$\gamma _{p}$	Probability of switching parameter	40
$\gamma _{e}$	Probability of entry parameter	-40

**Table 5:** Epidemiological model parameters

Symbol	Description	Value
$\phi _y$	Share of young agents in the population	0.15
$\phi _m$	Share of middle-aged agents	0.65
$\phi _o$	Share of old agents	0.2
$\pi ^i$	Probability of catching the normal disease	0.0012
$D^n_d$	Duration normal disease	4
	Susceptibility probability normal disease	0.1
$\sigma ^L$	Consumption shock to L-goods parameter (baseline and lower bound)	[1.65e$-$3,1.65e$-$4]
$\sigma ^B$	Consumption shock to B-goods parameter (baseline and lower bound)	[5.5e$-$4,5.5e$-$5]
$\pi ^h_y$	Share of young agents with serious symptoms	0.01
$\pi ^h_m$	Share of middle aged agents with serious symptoms	0.02
$\pi ^h_o$	Share of old agents with serious symptoms	0.525
	Total number of possible connections	449985000
	Number of permanent connections	29999
	Share of deactivated L-firms in lockdown	1/3
$d_{\max }^{locl}$	Lockdown maximum duration (weeks)	12
$\dot{ \mathcal {D}}_{lock}$	Lockdown activation threshold (new detected)	30
$\dot{ \mathcal {D}}_{end}$	Lockdown lifting threshold	12.5
$d^i$	Duration of epidemic disease (weeks)	$\Vert \mathcal {U}(3,5) \Vert$
*z*	Post-lockdown adjustment parameter	0.0775
	Share of connections under lockdown	0.25
	Share of work connections under lockdown	0.375
	Share of shop connections (out of weekly visitors)	1/3
	Share of shop connections under lockdown	(1/3)$\cdot$0.25
$c_{SD}$	Cost of distancing (in lockdown)	6 (-6)
$\iota$	Persistence of distancing index	0.725
$\beta$	Distancing effect on infection probability	1/3
$\rho _{c,\tau }$	Transmission rate (October to April)	0.07
$\rho _{c,\tau }$	Transmission rate (May to September)	0.04
$\overline{\mathcal {D}}_{c,SD}$	Social distancing threshold	5
$\textbf{N}$	Intensity of choice for social distancing	[0.05, 0.5, 1]
$h_1$	Health demand parameter	2
$h_2$	Health demand parameter	0.1
$h_3$	Death probability parameter (baseline and lower bound)	[0.0375,0.0125]
$\pi ^m$	Death probability (baseline and lower bound)	[0.0075,0.0025]
$\pi ^r$	Detection probability (baseline and upper bound)	[0.02,0.125]
$\gamma _{d}$	Adjustment of detection probability	0.0005
	Post-infection immunity duration (weeks)	$\mathcal {N}(52,2)$
$\mathcal {I}_{0}$	Number of initially infected	5
$\pi ^n$	Replacement probability of dead agents	0.0125

**Table 6:** Vaccine parameters

Description	Value
Coverage rate (initial value)	0.01
Weekly increments and upper bound of coverage rate	[0.001,0.05]
Vaccine efficacy w.r.t. contagion	0.80
Vaccine efficacy w.r.t. serious disease	0.95
Vaccine-induced immunity duration (weeks)	$\mathcal {N}(52,2)$

**Table 7:** Variant parameters

Description	Value
Number of initially infected with variant	5
Transmission rate of variant (October to April)	0.105
Transmission rate of variant (May to September)	0.095
Reduction factor distancing effect	0.75
Variant 1,2: reduction factor vaccine efficacy w.r.t contagion	[0.2,0.2]
Variant 1,2: reduction factor vaccine efficacy w.r.t. serious disease	[0,0.2]

## Footnotes

1 Mellacher (2022) uses a detailed epidemiological agent-based model to study the emergence and diffusion of mutations. The macroeconomic impact of these, however, is not explored.

2 In normal times, illness always ends with recovery, during an epidemic it may end either with recovery or with death.

3 Appendix D presents an experiment showing the effects of an increase in healthcare expenditure during an epidemic.

4 If a firm’s liquidity is negative but its equity is positive, it receives a transfer from the firm owner up to the financial wealth of the latter. If the firm’s liquidity is still negative after the transfer, the bank takes a loss equal to the negative balance and the firm’s deposit becomes zero, but the firm does not exit.

5 The most straightforward example of this phenomenon is the limited availability of beds in Intensive Care Units and equipment such as ventilators, particularly in the early stages of the pandemic.

6 In the baseline epidemic scenario the mean of this distribution – i.e., the average duration of natural immunity – is 52 weeks. In Appendix D.2 we experiment with varying durations of natural immunity.

7 As shown in Appendix D, an increase in healthcare expenditure has no significant effect on the number of infections, but considerably reduces the number of deaths resulting from this scenario.

8 As in the case of natural immunity we assume that the mean of this distribution – i.e., the average vaccine-induced immunity – is 52 weeks. In Appendix D.3 we experiment with varying durations of vaccine-induced immunity.

9 In the left panels of Fig. 5 we show the cumulative numbers of detected infections and fatalities since the beginning of the epidemic. The numbers in Table 1 are not comparable with those used to produce the figures, since the former do not include detected infected or deceased prior to the beginning of the vaccination campaign.

10 Numerically, the transmission rate of the original virus is 0.07 in the period October to April and 0.04 between May and September. We assume that the transmission rate of any variant is 0.105 (October to April) and 0.095 (May to September). The difference in transmission rates between seasons therefore shrinks from 0.03 to 0.01 in the presence of a variant.

11 We model the emergence of a variant as an exogenous shock to the epidemiological scenario with vaccination. In more sophisticated epidemiological agent-based models such as  Mellacher (2022), the emergence of variants is endogenous. While the endogenous emergence of variants is undoubtedly an appealing feature of a macro-epidemiological model, the exogeneity assumption is justified in our context because of the focus on a single region, Lombardy, within the context of a worldwide pandemic (indeed, all relevant variants of the SARS-COV2 virus discovered to date are held to have originated outside of Lombardy). Our modelling choice allows to better track the consequences of the variant, which is clearly identified as an exogenous shock with a pre-determined timing in the simulated model.

## Declarations

The authors state that

$\bullet$ this manuscript describes original work and is not under consideration for publication in any other journal;

$\bullet$ all authors approved the manuscript and this submission;

$\bullet$ research incorporated in this manuscript does not involve human participants or animals;

$\bullet$ our work proceeds within a theoretical and mathematical framework that must be analyzed by means of simulations. We do not analyse an empirical dataset but use publicly available data to calibrate the model. The simulations generate a set of artificial data. This artificial dataset is available from the corresponding author on reasonable request.
